# A novel route to a chiral building block for the preparation of cyclopentenyl carbocyclic nucleosides. Synthesis and anticancer activity of enantiomeric neplanocins A†

**DOI:** 10.1039/d0ra06394k

**Published:** 2020-08-27

**Authors:** Beata Łukasik, Maciej Mikina, Marian Mikołajczyk, Róża Pawłowska, Remigiusz Żurawiński

**Affiliations:** Division of Organic Chemistry, Centre of Molecular and Macromolecular Studies, Polish Academy of Sciences Sienkiewicza 112 90-363 Łódź Poland remzur@cbmm.lodz.pl +48-426803260; Division of Bioorganic Chemistry, Centre of Molecular and Macromolecular Studies, Polish Academy of Sciences Sienkiewicza 112 90-363 Łódź Poland

## Abstract

The synthesis of both enantiomers of 3-[(*tert*-butyldimethylsilyl)oxy]methyl-4,5-*O*-isopropylidenecyclopent-2-en-1-ol was accomplished in six steps based on optically inactive dimethyl *meso*-tartrate. This key intermediate in the synthesis of cyclopentenyl carbocyclic nucleosides was subsequently applied in the preparation of enantiomeric neplanocins A. The toxic effect of these compounds was investigated for a series of suspension and adherent cancer cell lines and normal human fibroblasts. (−)-Neplanocin A ((−)-NPA) was more toxic against all tested cancer cell lines than its dextrorotary counterpart. The highest toxicity with IC_50_ values of 7 and 10 μM was observed for the MOLT-4 and A431 cells, respectively. Moreover, (−)-NPA also induced apoptosis in A431 cell while this effect was not observed for (+)-NPA.

## Abstract 2

A novel route to both enantiomers of a cyclopentene building block was developed and applied to the synthesis of enantiomeric neplanocins A.

## Graphical abstract

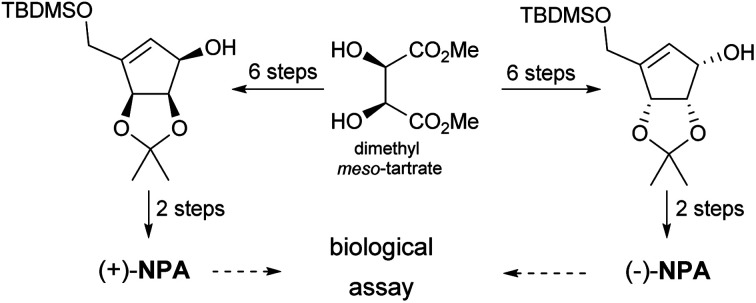

## Introduction

**Fig. 1:**
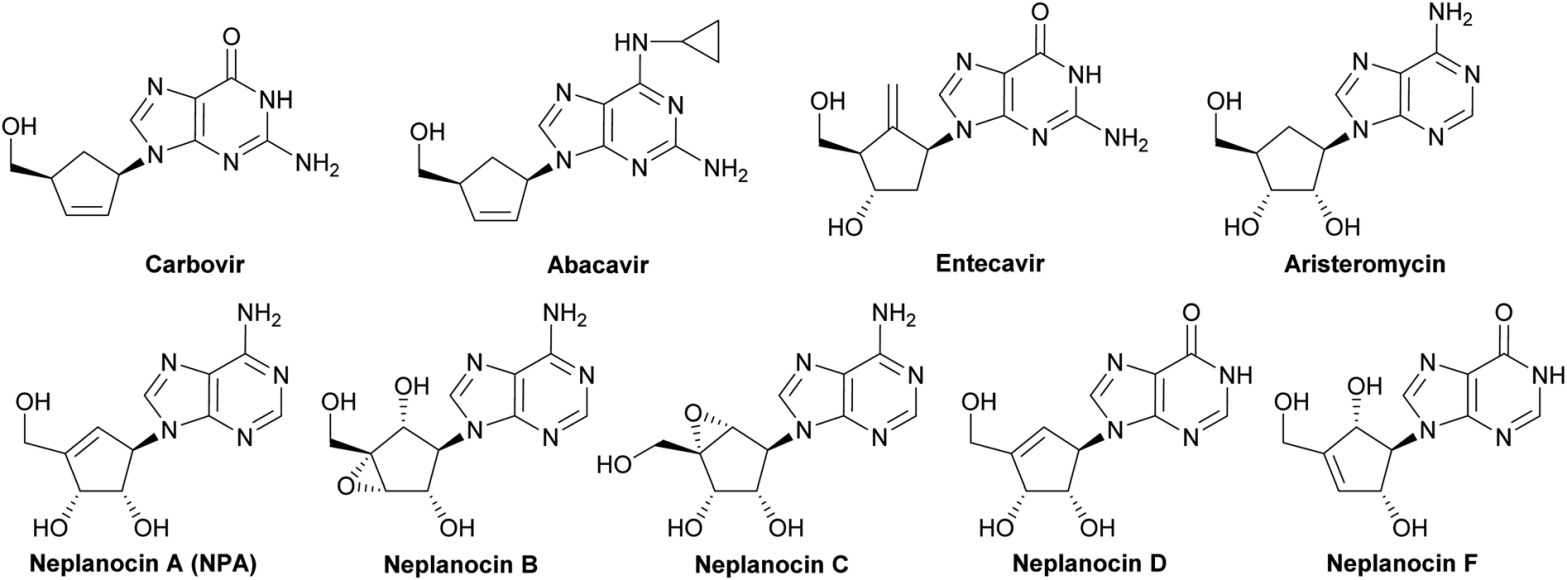
Selected carbocyclic nucleosides.

Carbocyclic nucleosides, namely nucleosides in which the sugar part is replaced by a carbocyclic moiety, constitute an important group of biologically active compounds.^1–3^ In spite of this structural modification, carbocyclic nucleosides are still recognized by the same enzymes as natural nucleosides exhibiting at the same time increased chemical stability and resistance *in vitro* to the degradative action of phosphorylases and hydrolases, due to the lack of the labile glycosidic bond.^4^ The class of carbocyclic nucleosides encompasses, among others, synthetic compounds with significant therapeutic properties such as carbovir^5^ and abacavir^6^ which have been approved for the treatment of the HIV infection, entecavir (inhibitor of hepatitis B virus polymerase),^7^ and those derived from natural sources and represented by aristeromycin^8^ and neplanocin family members (neplanocin A–D and F)^9^ (Fig. 1).

Especially, neplanocin A ((−)-NPA), isolated from the culture filtrate of the soil fungus *Ampullariella regularis*,^9–11^ emerged as a target of intense investigations of many research groups, mainly due to its broad spectrum of antiviral activity.^12–14^ It proved to be effective against numerous RNA and DNA viruses, particularly against vaccinia virus, vesicular stomatitis virus, parainfluenza virus type 3, reovirus type 1, human rotavirus and human immunodeficiency virus (HIV-1). Beside the strong antiviral activity, natural (−)-NPA also reveals weak antibacterial or antifungal properties^10^ and significant cytotoxicity against several cell types, including leukemic (L1210),^15^ breast,^16,17^ colon,^18^ prostate, liver, stomach and lung cancer cells.^19^ (−)-NPA is a potent inhibitor of histone H3-lysine79 (H3K79) methyltransferase^17^ and *S*-adenosylhomocysteine (AdoHcy) hydrolase^20^ that alters the *S*-adenosinemethionine-dependent methylation reactions and in consequence hampers the biosynthesis of cellular and viral rybonucleic acids and proteins.^21^ (−)-NPA also inhibits CSC as well as restricts migration and invasiveness of the breast cancer cells which are extremely important from the point of view of metastatic potential of a tumor.^17^ Although the mode of cytotoxic action of (−)-NPA is not completely clarified, it was reported that its toxicity may be resulted from the induction of signaling pathways leading to apoptosis.^17,18^

**Scheme 1:**
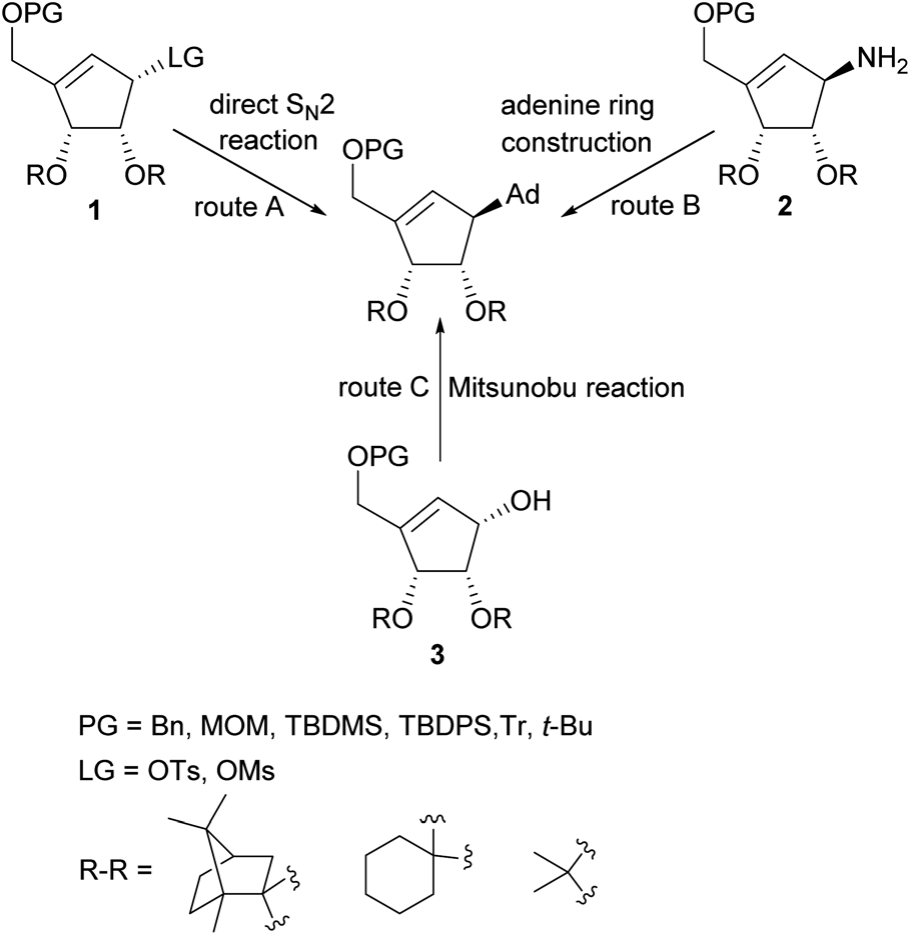
General strategies for the construction of NPA skeleton.

Several total syntheses of NPA in a racemic^22^ or optically active form^14,19,23–48^ were elaborated. These syntheses mainly involved three strategies for the construction of carbocyclic nucleoside skeleton (Scheme 1), which encompass: (i) direct S_N_2 reaction of an appropriate functionalized cyclopentenyl derivative 1, containing a good leaving group, with an adenine salt (route A); (ii) gradual assembling of the adenine framework based on cyclopentenylamine 2 (route B); (iii) the Mitsunobu reaction of protected tetrol 3 with adenine or its derivative (route C).

The later method, due to its simplicity, effectiveness and step economy, gained the highest popularity which resulted in the development of numerous approaches to these chiral cyclopentene derivatives. The key steps in their preparation included: palladium-catalyzed allylic rearrangement of the acetate moiety in the functionalized cyclopentane,^26^ application of the intramolecular Wittig^28^ and Horner–Wadsworth–Emmons reaction^38^ for the carbocyclic ring construction, stereospecific chloromethylation of chiral 4,5-dihydroxycyclopent-2-enone acetonide,^33^ intramolecular insertion of alkylidenecarbene to C–H bond,^34,47^ a lipase-mediated kinetic resolution of tricyclic Dielse–Alder adduct,^37^ stereoselective Michael–aldol tandem cyclization,^40^ zirconocene-mediated ring contraction of a vinyl-substituted pyranoside,^42^ intramolecular nitrone cycloaddition followed by N–O bond cleavage,^41^ cascade Knovenagel condensation–Horner olefination reaction,^44^ intramolecular Baylis–Hillman reaction leading to functionalized cyclopentene ring^45^ and intramolecular olefin metathesis using Furstner or second generation Grubbs catalyst.^43,46,48,49^ Most syntheses of these chiral key intermediates were based on a chiral pool such as d-ribose, d-glucose, l-tartaric acid or d-ribonolactone affording tetrol 3 stereoisomers leading to natural levorotary NPA. The synthesis of a second enantiomer often require other chiral starting material with an appropriate stereoconfiguration.

**Scheme 2:**
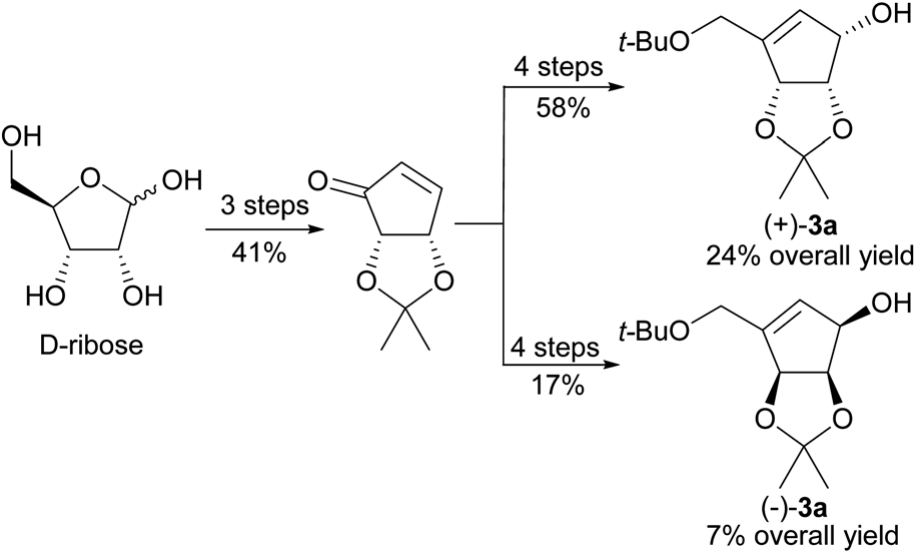
Synthesis of enantiomerically pure protected tetrols 3a from d-ribose.

Untill now, five total syntheses of unnatural NPA stereoisomer were reported^14,38,44,46,48^ (including the one in which a highly racemized product was obtained^38^), two of which were based on the common chiral or *meso* substrate for both enantiomeric NPAs. In 2001 Chu and coworkers disclosed the synthesis of enantiomeric neplanocines A from d-ribose (Scheme 2).^14^ The key enantiomeric intermediates (+)-3a and (−)-3a were prepared in a seven step reaction sequence, with three common steps, in 24% and 7% yield, respectively.

**Scheme 3:**
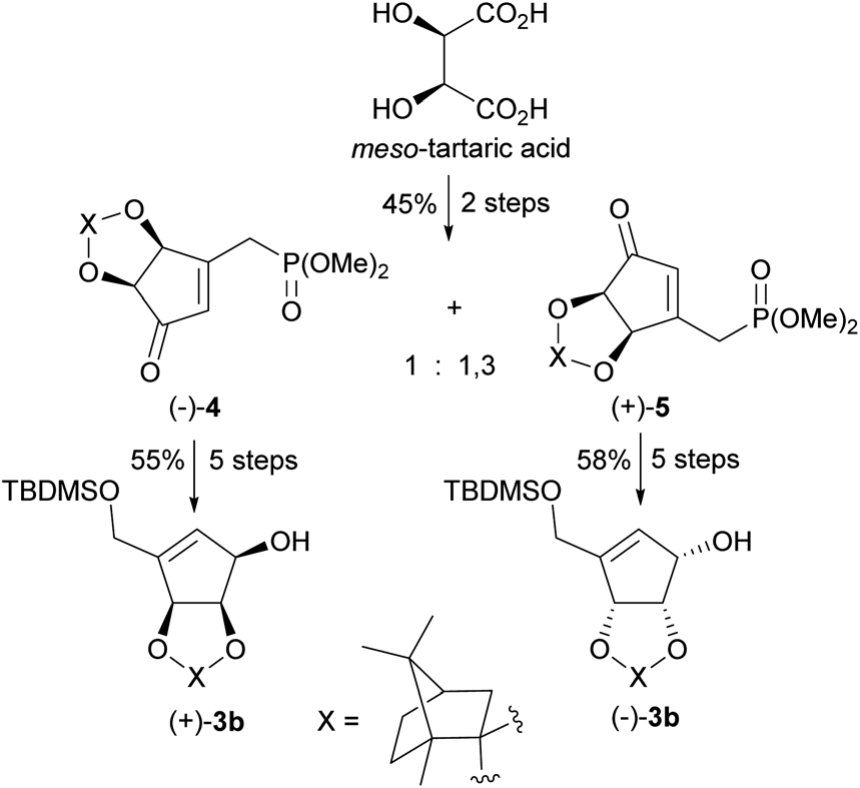
Synthesis of enantiomerically pure protected tetrols (−)-3b and (+)-3b from *meso*-tartaric acid.

In 2009 our group patented the synthesis of both enantiomers on NPA^44^ based on the developed by us diastereoisomeric camphor protected 3-[(dimethoxyphosphoryl)methyl]-4,5-dihydroxycyclopent-2-enones 4 and 5.^50^ These chiral cyclopentenone building blocks were prepared in two steps from *meso*-tartaric acid and subsequently converted in a five-step reaction sequence into enantiomeric alcohols (−)-3b and (+)-3b (Scheme 3). Although phosphonates 4 and 5 were also successfully applied in the syntheses of both enantiomers of anticancer cyclopentenone prostaglandin analogue TEI-9826 ^51^ and the two cross-conjugated derivatives of prostaglandin A and J series with neurotrophic activity,^52^ their preparation suffers from a low diastereoselectivity of the cyclopentenone ring formation and difficult separation.

In continuation and extension of our broad-term project on the application of phosphonate reagents and building blocks in the synthesis of biologically active compounds,^53^ and to overcome the limitation of diastereoisomeric phosphonates 4 and 5, we report herein a new approach to the synthesis of both enantiomers of 3-[(*tert*-butyldimethylsilyl)oxy]methyl-4,5-*O*-isopropylidenecyclopent-2-en-1-ol (3c) and their application in the synthesis of (+)- and (−)-NPA. Because, to the best of our knowledge, anticancer properties of (+)-NPA have never been reported, in the present paper we also present the results of comparative biological studies of both enantiomers of NPA. The toxic effect of (+)- and (−)-NPA were investigated in the selected, cancer cell types including breast cancer (MDA-MB-231 and MCF-7 cells), epidermoid carcinoma (A431), human glioblastoma (U87-MG), T-cell acute lymphoblastic leukemia (MOLT-4), and in normal human fibroblasts. Additionally, the ability to induce a programmed cell death was estimated based on the two techniques: DAPI staining and measurements of caspase-3 and caspase-7 activity level.

## Results and discussion

### Synthesis on enantiomeric NPAs

**Scheme 4:**
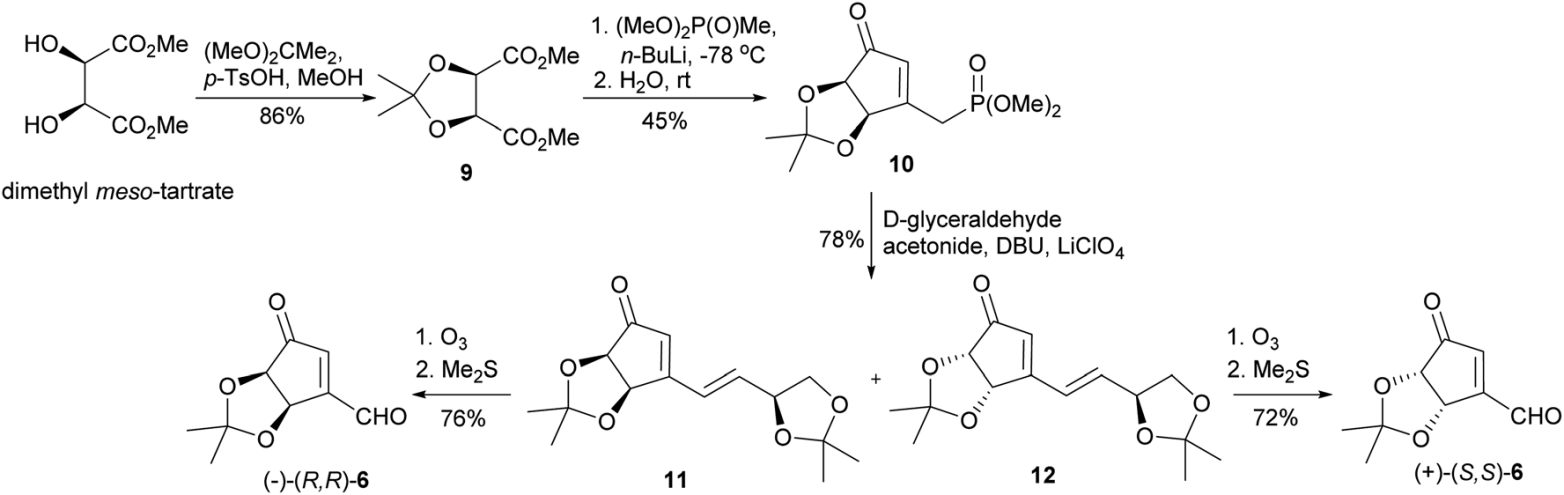
Synthesis of enantiomeric ketoaldehydes 6.

Our synthesis of both enantiomers of NPA started with preparation of enantiomerically pure ketoaldehydes 6. These compounds were synthesized according to the recently reported by us four-step protocol^54^ based on the commercially available dimethyl *meso*-tartrate (also easily accessible from *meso*-tartaric acid according to a known literature procedure^54^) as a starting material (Scheme 4). The first step of this synthesis involved the protection of two hydroxy groups in dimethyl *meso*-tartrate and was achieved by its acid catalyzed reaction with 2,2-dimethoxypropane. The obtained acetonide 9 was subjected to the reaction with lithium salt of dimethyl methylphosphonate to give phosphonate 10 in a moderate yield. The olefination reaction of 10 with d-glyceraldehyde acetonide conducted under the condition of the Horner reaction gave an easily separable equimolar mixture of diastereoisomeric dienones 11 and 12. Selective ozonolysis of the exocyclic carbon–carbon double bond in 11 and 12 afforded enantiomerically pure ketoaldehydes (−)-(*R*,*R*)-6 and (+)-(*S*,*S*)-6 in 76% and 72% yield, respectively.

**Scheme 5:**
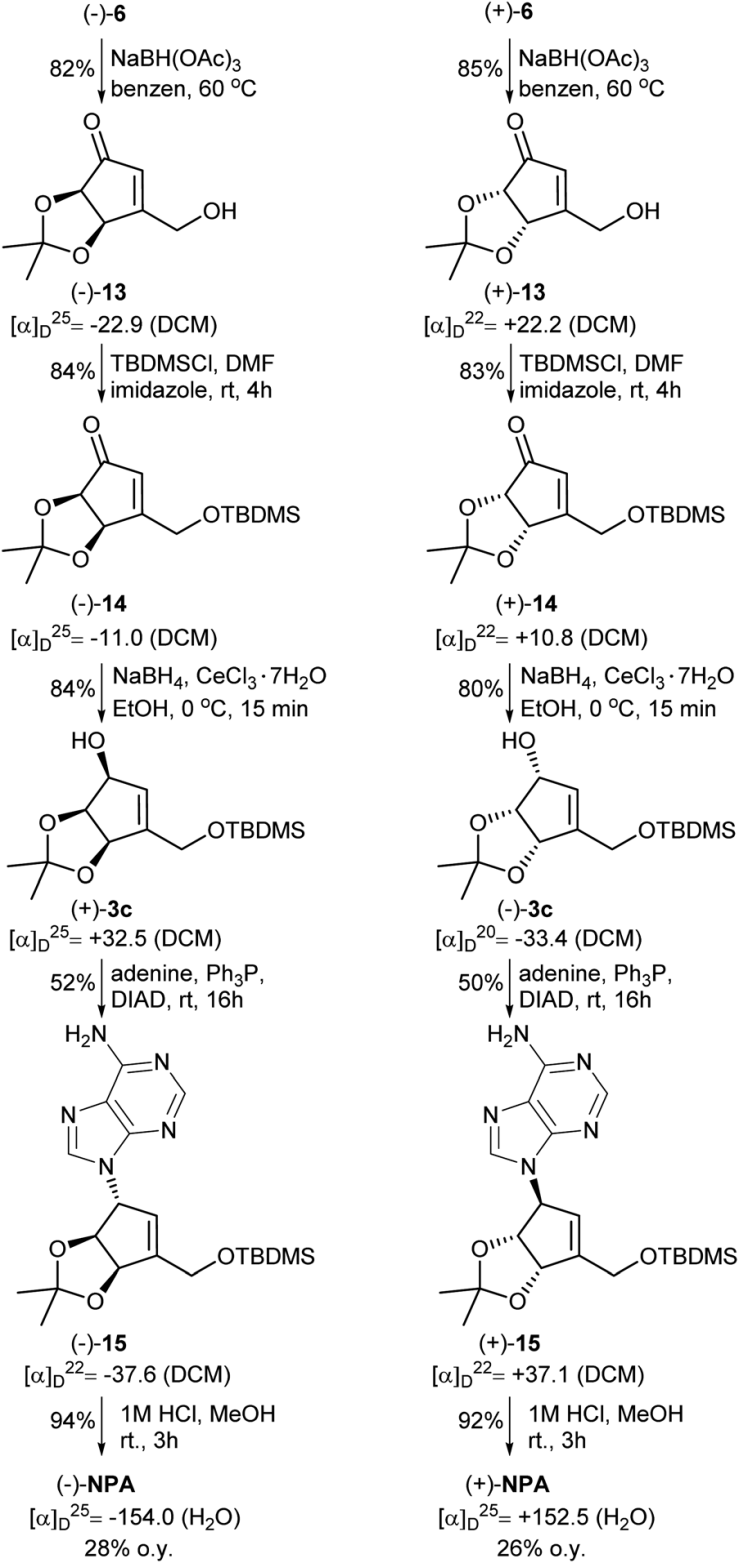
Synthesis of enantiomeric neplanocins A from ketoaldehydes 6.

The next part of the synthesis of neplanocin A enantiomers is outlined in Scheme 5. Selective reduction of the formyl group in α,β-unsaturated ketoaldehydes 6 was accomplished by the treatment of these compounds with sodium triacetoxyborohydride in hot benzene. In the next step, the resulting 3-hydroxymethylcyclopentenones 13 were treated with TBDMSCl in the presence of imidazole to give the corresponding silyl ethers 14. The reduction of the carbonyl group in 14 by sodium borohydride in the presence of cerium(iii) chloride afforded desired protected (−)- and (+)-tetrols 3c in which the three oxygen atoms attached to the cyclopentene ring were in *cis* relation. Replacement of the hydroxyl group in tetrols 3c with adenine was achieved under the Mitsunobu reaction conditions. Deprotection of thus obtained carbocyclic nucleosides 15 conducted under the acidic conditions led to the formation of (−)- and (+)-NPA in 28% and 26% overall yield from enantiomeric aldehydes (−)-6 and (+)-6, respectively. The optical rotation, spectroscopic and physical data of enantiomeric neplanocins A so obtained were in agreement with those already given in the literature.^10,11^

**Scheme 6:**
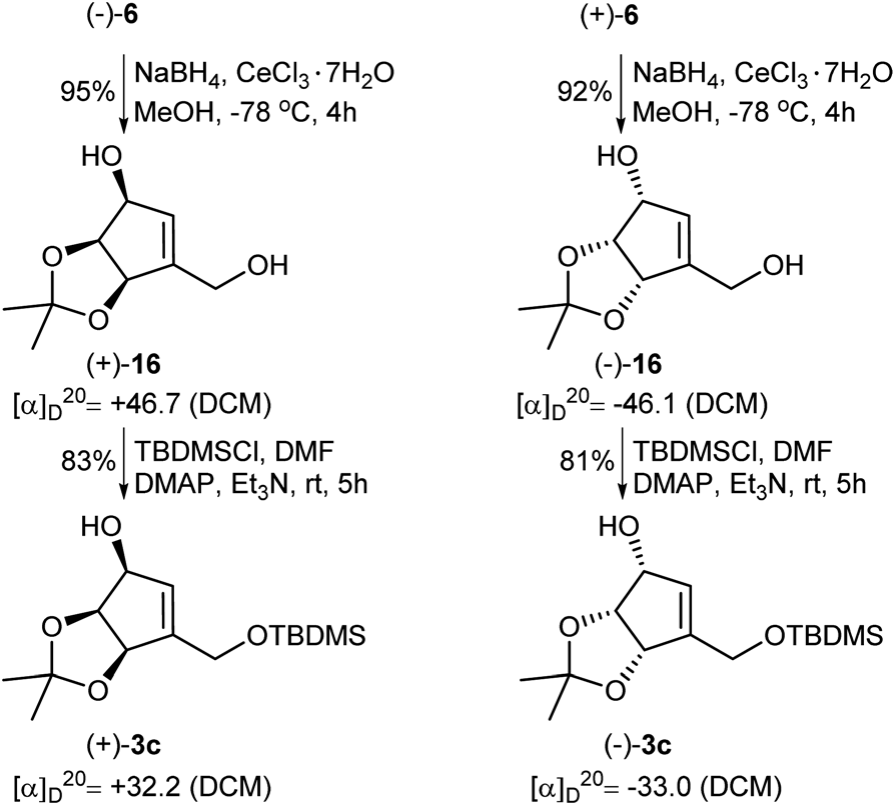
Transformation of enantiomeric ketoaldehydes 6 into the protected tetrols 3c.

In the course of our investigations on the synthesis of NPA, a shorter and more efficient protocol for the preparation of protected tetrols 3c from ketoaldehydes 6 was also elaborated (Scheme 6). Thus, in the first step two carbonyl groups in ketoaldehydes 6 were reduced under the Luche reduction conditions affording the corresponding diols 16 in a high yield. The second step consisted in a selective protection of a primary hydroxyl group in diols 16 as a *tert*-butyldimethyl silyl ether to give protected, enantiomerically pure tetrols 3c.

**Scheme 7:**
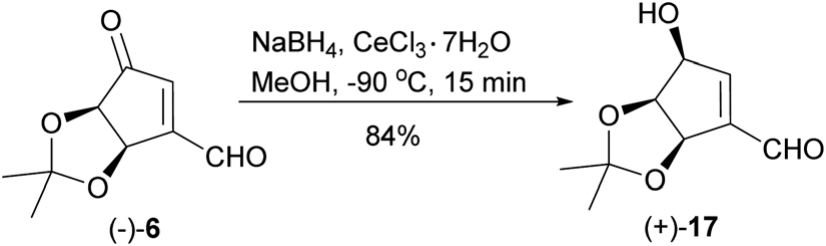
Chemo- and stereoselective reduction of ketoaldehyde (−)-6 into hydroxyaldehyde (+)-17.

When the reduction of ketoaldehyde (−)-6 was conducted at −90 °C in methanol, hydroxyaldehyde (+)-17 was isolated as the main product in 84% yield (Scheme 7). The absolute configuration of the newly created stereogenic center was confirmed by a transformation of this product into diol (+)-16 of an already known stereoconfiguration. The outcome of this reaction can be rationalized assuming deactivation of the aldehyde group by the formation of hemiacetal with methanol followed by the attack of alkoxyborohydride on the carbonyl group, which takes place from the less sterically hindered side (opposite to the *cis*-diol moiety) leading to hydroxyaldehyde (+)-17 with three oxygen atoms adjacent to the cyclopentene ring in *cis* orientation.

### Biological assays

#### Cytotoxicity

Both enantiomers of neplanocin A were investigated for their toxicity against the selected cancer cells. Simultaneously, the impact of these compounds on the normal human cells was also investigated. The results obtained showed the cytotoxic effect of (−)-NPA against all tested cancer cell lines, both suspension (MOLT-4) and adherent (MDA-MB-231, MCF-7, A431, U87-MG) (Table 1). Particularly potent antitumor activity of (−)-NPA was observed for A431 and MOLT-4 cells (IC_50_ value up to 10 μM). The (+)-NPA was much less toxic than its levorotatory counterpart against all the tested cancer cells and almost non-toxic for the MCF-7 cells at a concentrations up to 1 mM. Both enantiomers of NPA displayed cytotoxic selectivity between the cancer cell lines and normal human cells. Contrary to normal fibroblasts for which determination of IC_50_ for a concentration up to 1 mM after 48 h incubation time was not possible for both enantiomeric neplanocins A, for all cancer cells a strong correlation between absolute configuration of enantiomers and anticancer activity was observed.

**Table 1:** The cytotoxicity of (+)- and (−)-NPA after 48 hours incubation – IC_50_ values

Compound	Fibroblasts	MDA-MB-231	MCF-7	A431	U87-MG	MOLT-4
(+)-NPA	>1 mM	880 μM	>1 mM	330 μM	650 μM	500 μM
(−)-NPA	>1 mM	200 μM	55 μM	10 μM	40 μM	7 μM

Enantioselectivity of nucleotides and nucleosides recognizing enzymes is a well-known phenomenon related to the orientation of the amino acid chains in the folded molecule. It was demonstrated, that d- and l-enantiomers of adenosine triphosphate may exhibit partially different biological properties, however some cellular and viral enzymes may recognize both of them.^55^NPA as a carbocyclic analog of adenosine may interact with similar targets, as its natural ribonucleotidic counterpart. It was shown, that the conformational changes in both, sugar (north/south) and nucleobase (*syn*/*anti*) part of the neplanocin derivatives may contribute to the differences in their antiviral activity.^56–58^ On the other hand, both enantiomers of 1′,6′-isoneplanocin turned out to be inactive as inhibitors of *S*-adenosylhomocysteine hydrolase (SAHase) and for selected DNA and RNA viruses.^58^

#### Induction of apoptosis

**Fig. 2:**
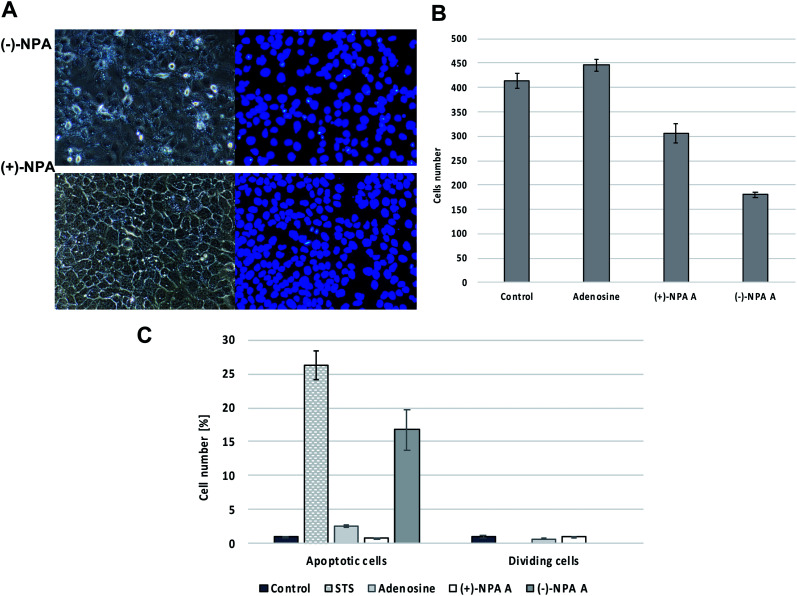
The effect of neplanocin A enantiomers on morphology and multiplicity of A431 cells after 18 hour treatment at 100 μM concentration, (A) fluorescent microscopic images of cells in phase contrast (left) and DAPI filter (right), (B) the average cells number per the field of view, (C) the number of apoptotic and cell division events in the field of view after cells treatment with tested compounds.

In order to identify the cell death pathway, DAPI (4′,6-diamidino-2-phenylindole) cell staining was performed. DAPI binds strongly to adenine–thymine rich regions in DNA staining cell nuclei and thus enabling visualization of changes occurring in them during apoptosis or the cell division, such as the formation of apoptotic bodies.^59,60^ Microscopic observation revealed the decreased number of A431 cells in the culture after 18 h treatment with (−)-NPA at 100 μM concentration in comparison to the control cells (cells incubated with DMSO) as well as to the cells treated with adenosine. In the case of (+)-NPA the impact on the cell population was smaller under the same experimental conditions (Fig. 2A and B). These observations were compatible with the MTT results, which also indicated on the lower toxicity of (+)-NPA in comparison to its natural enantiomer (Table 1).

Based on the differences in morphology of apoptotic and normal nuclei (presence of apoptotic bodies or highly condensed nuclear chromatin, respectively), the stained cells were easily distinguished. The significant changes in the cell morphology as well as an increased number of the apoptotic cells were observed in the culture after 18 h treatment with (−)-NPA and in the positive control (cells treated with staurosporin which is the strong inducer of the programmed cell death) (Fig. 2A and C). This result indicates that (−)-NPA cytotoxicity in the A431 cells is connected with the induction of apoptosis and is in agreement with the previous observations referring to a different cancer model.^17,18^ The similar effect for (+)-NPA was not observed. The DAPI staining method was also applied to assess the number of dividing cells. The cell division process was detected only for the cultures treated with adenosine and (+)-NPA. In the case of (−)-NPA and staurosporin none of the cell division events were noticed (Fig. 2C).

**Fig. 3:**
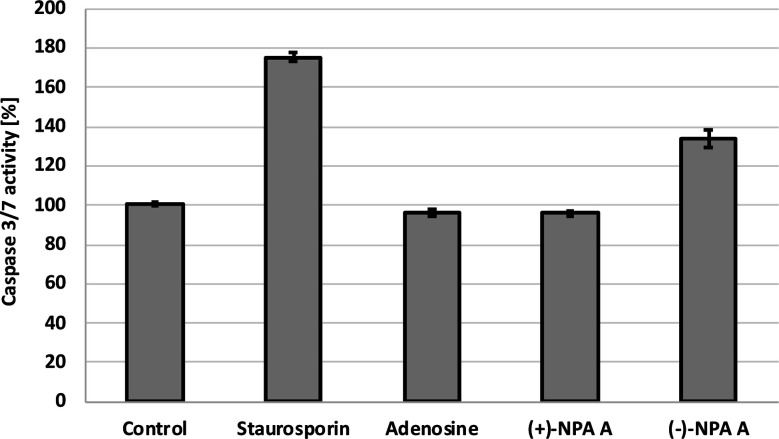
The activity level of caspase-3/7 in the A431 cells after 18 h incubation with compounds at 100 μM concentration. Staurosporin (5 μM) was used as a positive control.

Additional evidence for induction of apoptotic process by (−)-NPA in A431 cells was given by the measurement of change in the caspase-3/7 activity with respect to the control cells. Caspases are enzymes, which are activated during apoptosis and their activity can be a measure of this process. The results obtained confirmed the ability to initiate the programmed cell death by (−)-NPA, whereas under the same conditions dextrorotatory enantiomer was not active (Fig. 3). This difference in the mode of action between enantiomeric neplanocins A may result from the dissimilar interactions with molecular targets which affect the regulation of molecular signaling pathways. This phenomenon will be explored in further research.

## Conclusions

In conclusion, a new route to enantiomerically pure (+)- and (−)-3-[(*tert*-butyldimethylsilyl)oxy]methyl-4,5-*O*-isopropylidenecyclopent-2-en-1-ols (3c) was elaborated based on optically inactive dimethyl *meso*-tartrate. The advantage of this approach is that it gives an easy access to enantiomeric (+)- and (−)-3c which are commonly used as chiral building blocks not only in the synthesis of neplanocin A but also other cyclopentenyl carbocyclic nucleosides. Such an access to both enantiomers of the investigated compounds is especially important from the point of view of structure-activity relationship studies. The current method compares favorably in term of its convenience, the overall yield and the step economy with respect to the previously reported protocols based on the common starting material for both enantiomers.^14,44^ Based on chiral intermediates 3c the synthesis of both enantiomers of NPA was accomplished and the cytotoxic activity of these compounds was investigated in the model of human glioblastoma, epidermoid, lymphoblastic leukemia and breast cancer cells, as well as in normal human fibroblasts. Although previous reports have shown that (−)-NPA induces apoptosis in some cancer cell lines, so far (+)-NPA remained unexplored with regard to its anticancer activity. We found that (+)-NPA was much less toxic against all tested lines, and contrary to its enantiomer did not induce apoptosis in the A431 cancer cells. These results indicate the strong correlation between antitumor activity and stereoconfiguration in neplanocins A and can be a suggestion of structural requirements for biological activity of other neplanocin A derivatives and cyclopentenyl carbocyclic nucleosides.

## Experimental

### General remarks

Unless stated otherwise, all reactions with air and water sensitive compounds were carried out under an argon atmosphere using freshly distilled dry solvents. All glassware was dried prior to use by heating under vacuum. Commercial grade reagents and solvents were used without further purification except as indicated below. THF was distilled from Na/benzophenone prior to use. Triethylamine was dried under reflux over calcium hydride, distilled and stored over activated 3A molecular sieves. Benzene was dried by refluxing over sodium and distillation. Dry DMF was prepared by drying over 4A molecular sieves followed by vacuum distillation. Thin layer chromatography (TLC) was conducted on Silica Gel 60 F254 TLC purchased from Merck. Column chromatography was performed using Merck silica gel (70–230 mesh). NMR spectra were recorded on Bruker DRX 500 and Bruker Avance III 600 spectrometers. ^1^H, ^13^C chemical shifts are reported relative to the residual proton resonance in the deuterated solvents. All chemical shifts (*δ*) are given in ppm and the coupling constants (*J*) in Hz. HRMS measurements were performed on a Finnigan MAT 95 or Waters Synapt HDMS mass spectrometer. Optical rotations were measured using a Perkin-Elmer MC 241 photopolarimeter. Melting and boiling points are uncorrected.

#### (−)-(*R*,*R*)-4,5-Dihydroxy-3-(hydroxymethyl)cyclopent-2-enone acetonide ((−)-13)

To a stirred suspension of sodium borohydride (0.301 g, 7.9 mmol) in dry benzene (40 mL) acetic acid (1.551 g, 0.026 mol) in dry benzene (5 mL) was slowly added. The mixture was refluxed for 0.5 h and transferred to a solution of aldehyde (−)-6 (0.181 g, 0.994 mmol) in dry benzene (2 mL). After 5 min of gentle heating the reaction mixture was cooled down to room temperature and filtered through a silica gel pad. The solvent was evaporated and a crude mixture was purified by column chromatography (petroleum ether/acetone 2 : 1) to give hydroxyketone (−)-13 (0.141 g, 82%) as a colorless solid. Mp 63–64 °C; [*α*]^25^_D_ = −22.9 (*c* 1 in CH_2_Cl_2_); ^1^H NMR (500 MHz, CDCl_3_): *δ* 6.15 (s, 1H, C=C*H*), 5.11 (d, *J* = 5.0, 1H, OC*H*C(O)), 4.68 (d, *J* = 18.5, 1H, CH_2_OH), 4.52 (d, *J* = 18.5, 1H, CH_2_OH), 4.49 (d, *J* = 5.0, 1H, OC*H*–CCH_2_), 2.63 (s, 1H, O*H*), 1.38 (s, 6H, C*H*_3_). ^13^C NMR (126 MHz, CDCl_3_): *δ* 201.97 (*C*O), 176.74 (*C*CH_2_), 127.46 (C=*C*H), 115.59 ((CH_3_)_2_*C*), 77.89 (O*C*H–C–CH_2_), 77.79 (O*C*H–C(O)), 60.94 (*C*H_2_), 27.28 (*C*H_3_), 26.01 (*C*H_3_). HRMS (EI) calcd for C_9_H_12_O_4_ 184.0736, found 184.0729. C_9_H_12_O_4_ (184.19): calcd C 58.69, H 6.57; found C 58.73, H 6.61.

#### (+)-(*S*,*S*)-4,5-Dihydroxy-3-(hydroxymethyl)cyclopent-2-enone acetonide ((+)-13)

According to the procedure described above aldehydoketone (+)-6 (130 mg, 0.714 mmol) was transformed into hydroxyketone (+)-13 (113 mg, 85%).[*α*]^22^_D_ = + 22.2 (*c* 1.7 in CH_2_Cl_2_). C_9_H_12_O_4_ (184.19): calcd C 58.69, H 6.57; found C 58.43, H 6.84.

#### (−)-(4*R*,5*R*)-3-[(*tert*-Butyldimethylsilyl)oxy]methyl]-4,5-*O*-izopropylidenecyklopent-2-enon ((−)-14)

A solution of hydroxyketone (−)-13 (141 mg, 0771 mmol), TBDMSCl (231 mg, 1.54 mmol) and imidazole (210 mg, 3.08 mmol) in DMF (1.5 mL) was stirred at room temperature for 4 h. Saturated solution of NaHCO_3_ was added and the mixture was extracted with dichloromethane (4 × 20 mL). Combined organic fractions were washed with brine and dried over anhydrous Na_2_SO_4_. After evaporation of DCM the residue was subjected to column chromatography (petroleum ether/acetone 8 : 1) to afford (−)-14 (193 mg, 84%) as a colorless liquid. *R*_f_ = 0.34 (petroleum ether/acetone 8 : 1). [*α*]^25^_D_ = −11.0 (*c* 1.0 in CH_2_Cl_2_). ^1^H NMR (600 MHz, CDCl_3_): *δ* 6.16 (s, 1H, C=C*H*), 5.06 (d, *J* = 5.6, 1H, OC*H*–C(O)), 4.66 (d, *J* = 18.8, 1H, C*H*_2_O), 4.51 (d, *J* = 5.6, 1H, OC*H*–CCH_2_), 4.47 (d, *J* = 18.7, 1H, C*H*_2_O), 1.40 (s, 6H, C*H*_3_), 0.92 (s, 9H, (C*H*_3_)_3_C), 0.10 (s, 6H, (C*H*_3_)_2_Si). ^13^C NMR (151 MHz, CDCl_3_): *δ* 201.69 (*C*O), 177.25 (C=*C*CH_2_), 127.78(C=*C*H), 115.46 ((CH_3_)_2_*C*), 78.07 (O*C*H–CCH_2_), 77.67 (O*C*H–C(O)), 61.59 (*C*H_2_), 27.43 (*C*H_3_), 26.20 (*C*H_3_), 25.74 (3C, (*C*H_3_)_3_C), 18.28 ((CH_3_)_3_*C*Si), −5.49 (*C*H_3_Si), −5.00 (*C*H_3_Si). HRMS (ESI+) calcd for C_15_H_26_O_4_SiNa [M + Na]^+^ 321.1498, found 321.1501.

#### (+)-(4*S*,5*S*)-3-[(*tert*-Butyldimethylsilyl)oxy]methyl]-4,5-*O*-izopropylidenecyklopent-2-enon ((+)-14)

According to the procedure described above the reaction of (+)-13 (132 mg, 0.717 mmol) with TBDMSCl (215 mg, 1.433 mmol) and imidazole (195 mg, 2.866 mmol) in DMF (1.5 mL) afforded silyl ether (+)-14 (176 mg, 83%) as a colorless liquid. [*α*]^22^_D_ = +10.8 (*c* 1.7 in CH_2_Cl_2_). HRMS (ESI+) calcd for C_15_H_26_O_4_SiNa [M + Na]^+^ 321.1498, found 321.1495.

#### (+)-(1*S*,4*R*,5*S*)-3-[(*tert*-Butyldimethylsilyl)oxy]methyl-4,5-*O*-isopropylidenecyclopent-2-en-1-ol ((+)-3c) from (−)-14

To a stirred solution of silyl ether (−)-14 and CeCl_3_·7H_2_O (187 mg, 0.502 mmol) in ethanol (4 mL) at 0 °C was slowly added a solution of sodium borohydride (9.5 mg, 0.251 mmol) in ethanol (1 mL). After 15 min saturated aqueous solution of NH_4_Cl was added and ethanol was evaporated under reduced pressure. The residue was extracted with dichloromethane (4 × 10 mL). The combined organic layers were washed with brine, dried over anhydrous Na_2_SO_4_ and concentrated under reduced pressure. The crude material was purified by column chromatography (petroleum ether/acetone 10 : 1) affording alcohol (+)-3c (125 mg, 84%) as a colorless liquid. *R*_f_ = 0.56 (petroleum ether/acetone 8 : 1). [*α*]^25^_D_ = +32.5 (*c* 0.8 in CH_2_Cl_2_). ^1^H NMR (500 MHz, CDCl_3_): *δ* 5.72 (s, 1H, C=C*H*), 4.89 (d, *J* = 5.6, 1H, OC*H*–C(O)), 4.75 (t, *J* = 5.5, 1H, OC*H*–CCH_2_), 4.58–4.50 (m, 1H, C*H*OH), 4.34 (d, *J* = 15.1, 1H, C*H*_2_OSi), 4.23 (d, *J* = 15.1, 1H, C*H*_2_OSi), 2.67 (d, *J* = 10.1, 1H, O*H*), 1.41 (s, 3H, C*H*_3_), 1.38 (s, 3H, C*H*_3_), 0.90 (s, 9H, (C*H*_3_)_3_C), 0.07 (s, 3H, C*H*_3_Si), 0.07 (s, 3H, C*H*_3_Si). ^13^C NMR (126 MHz, CDCl_3_): *δ* 145.82 (*C*CH_2_), 129.46 (C=*C*H), 112.70 ((CH_3_)_2_*C*), 82.97 (*C*HOH), 78.18 (O*C*H–CCH_2_), 73.47 (O*C*H–C(O)), 60.17 (*C*H_2_), 27.88 (*C*H_3_), 26.87 (*C*H_3_), 26.10 (3C, (*C*H_3_)_3_C), 18.60 ((CH_3_)_3_*C*), −5.18 (*C*H_3_Si), −5.23 (*C*H_3_Si). HRMS (ESI+) calcd for C_15_H_28_O_4_SiNa [M + Na]^+^ 323.1655, found 323.1662.

#### (−)-(1*R*,4*S*,5*R*)-3-[(*tert*-Butyldimethylsilyl)oxy]methyl-4,5-*O*-isopropylidenecyclopent-2-en-1-ol ((−)-3c) from (+)-14

By analogy to the procedure describe above enone (+)-14 (173 mg, 0.579 mmol) was transformed into alcohol (−)-3c (140 mg, 80%). [*α*]^20^_D_ = −33.4 (*c* 0.9 in CH_2_Cl_2_). HRMS (ESI+) calcd for C_15_H_28_O_4_SiNa [M + Na]^+^ 323.1655, found 323.1660.

#### (+)-(1*S*,4*R*,5*S*)-3-[(*tert*-Butyldimethylsilyl)oxy]methyl-4,5-*O*-isopropylidenecyclopent-2-en-1-ol ((+)-3c) from diol (+)-16

A solution of diol (+)-16 (74 mg, 0.40 mmol), TBDMSCl (66 mg, 0.44 mmol), triethylamine (45 mg, 0.44 mmol) and 4-dimethylaminopyridine (5 mg, 0.04 mmol) in DMF (0.7 mL) was stirred at room temperature for 5 h. Then, a saturated aqueous solution of NaHCO_3_ was added and the mixture was extracted with hexane/ethyl acetate 1 : 1 (4 × 15 mL). The combined organic layers were washed with brine and dried over Na_2_SO_4_. Concentration followed by silica gel chromatography (petroleum ether/acetone 8 : 1) afforded alcohol (+)-3c (99 mg, 83%) as a colorless liquid. [*α*]^20^_D_ = +32.2 (*c* 1.1 in CH_2_Cl_2_).

#### (−)-(1*R*,4*S*,5*R*)-3-[(*tert*-Butyldimethylsilyl)oxy]methyl-4,5-*O*-isopropylidenecyclopent-2-en-1-ol ((−)-3c) from diol (−)-16

According to the above procedure, diol (−)-16 (62 mg, 0.333 mmol) was reacted with TBDMSCl (55 mg, 0.366 mmol) in the presence of triethylamine (37 mg, 0.366 mmol) and 4-dimethylaminopyridine (4 mg, 3.3 μmol) in anhydrous DMF (0.6 mL) to give alcohol (−)-3c (81 mg, 81%). [*α*]^20^_D_ = −33.0 (*c* 1.1 in CH_2_Cl_2_).

#### (−)-(1*R*,4*R*,5*S*)-1-(6-Amino-9*H*-purin-9-yl)-3-{[(*tert*-Butyldimethylsilyl])oxy]methyl}-4,5-*O*-isopropylidenecyclopent-2-en ((−)-15)

To a stirred solution of triphenylphosphine (273 mg, 1.04 mmol) in THF (5 mL) diisopropyl azodicarboxylate (DIAD) (209 mg, 204 μl, 1.04 mmol) was added dropwise at 0 °C. After 30 min a solution of alcohol (+)-3c (125 mg, 0.042 mmol) in THF (2.5 mL) was added and stirring was continued for an additional 15 min. Then, adenine (140 mg, 1.04 mmol) was added, cooling batch was removed and the reaction mixture was stirred overnight at room temperature. The solvent was evaporated *in vacuo*, and the residue was subjected to column chromatography (petroleum ether/acetone 1 : 1) to yield (−)-15 (90 mg, 52%) as a colorless liquid. *R*_f_ = 0.5 (petroleum ether/acetone 1 : 1). [*α*]^25^_D_ = −37.6 (*c* 0.3 in CH_2_Cl_2_). ^1^H NMR (600 MHz, CDCl_3_): *δ* 8.40 (s, 1H, C*H*=N (adenine)), 7.68 (s, 1H, C*H*=N (adenine)), 5.78 (s, 1H, C*H*N), 5.66 (s, 2H, N*H*_2_), 5.59 (s, 1H, C*H*=C), 5.30 (d, *J* = 5.6, 1H, OC*H*–CN), 4.71 (d, *J* = 5.7, 1H, OC*H*–CCH_2_), 4.45 (d, *J* = 16.1, 1H, C*H*_A_H_B_O), 4.41 (d, *J* = 16.2, 1H, CH_A_*H*_B_O), 1.48 (s, 3H, C*H*_3_C), 1.35 (s, 3H, C*H*_3_C), 0.92 (s, 9H, (C*H*_3_)_3_CSi), 0.11 (s, 6H, (C*H*_3_)_2_Si); ^13^C NMR (151 MHz, CDCl_3_): *δ* 154.88 (*C*H=N (adenine)), 152.84 (*C*=C (adenine)), 152.03 (C=*C* (adenine)), 149.47 (C=*C*–CH_2_), 138.07 (*C*H=N (adenine)), 120.62 (*C*HN), 119.69 (*C*NH_2_ (adenine)), 112.24((CH_3_)_2_*C*), 84.40 (O*C*H–CCH_2_), 83.10 (O*C*H–CHN), 63.94 (*C*=C–CH_2_), 59.93 (*C*H_2_), 26.94 (*C*H_3_), 25.47 (*C*H_3_), 25.38 (3C, (*C*H_3_)_3_C), 17.93 ((CH_3_)_3_*C*), −5.86 (*C*H_3_Si), −5.89 (*C*H_3_Si). HRMS (ESI+) calcd for C_20_H_31_O_3_N_5_Si [M + H]^+^ 418.2274, found 418.2277.

#### (+)-(1*S*,4*S*,5*R*)-1-(6-Amino-9*H*-purin-9-yl)-3-{[(*tert*-butyldimethylsilyl])oxy]methyl}-4,5-*O*-isopropylidenecyclopent-2-en ((+)-15)

According to the above procedure alcohol (−)-3c (140 mg, 0.466 mmol) upon treatment with DIAD (235 mg, 1.16 mmol), triphenylphosphine (305 mg, 1.16 mmol) and adenine (157 mg, 1.16 mmol) was transformed into (+)-15 (97 mg, 50%). [*α*]^25^_D_ = +37.1 (*c* 0.3 in CH_2_Cl_2_). HRMS (ESI+) calcd for C_20_H_31_O_3_N_5_Si [M + H]^+^ 418.2274, found 418.2276.

#### (+)-(1*S*,4*R*,5*S*)-3-Hydroksymetyl-4,5-*O*-isopropylidenecyclopent-2-en-1-ol ((+)-16)

A solution of ketoaldehyde (−)-6 (100 mg, 0.54 mmol) and cerium(iii) chloride heptahydrate (818 mg, 2.19 mmol) in methanol (7 mL) was cooled down to −78 °C and sodium borohydride (63 mg, 1.64 mmol) was added in portions during 20 min. A mixture was stirred for 4 h and a saturated aqueous solution of NH_4_Cl was added, the cooling batch was removed and the mixture was warmed to room temperature. Methanol was evaporated at a reduced pressure and the residue was extracted with CHCl_3_ (3 × 30 mL). The combined organic extracts were dried over Na_2_SO_4_, concentrated under vacuum and the crude material was purified by column chromatography (petroleum ether/acetone 2 : 1) to yield diol (+)-16 (97 mg, 95%) as a waxy white solid. *R*_f_ = 0.18 (petroleum ether/acetone 2 : 1). [*α*]^22^_D_ = +46.7 (*c* 0.9 in CH_2_Cl_2_). ^1^H NMR (500 MHz, CDCl_3_): *δ* 5.84–5.60 (m, 1H, C*H*=C), 4.98 (d, *J* = 5.5, 1H, OC*H*–CCH_2_), 4.78 (t, *J* = 5.5, 1H, OC*H*–CHOH), 4.61–4.52 (m, 1H, C*H*OH), 4.34 (d, *J* = 14.0, 1H, C*H*_A_H_B_), 4.28 (dd, *J* = 14.3, 5.1, 1H, CH_A_*H*_B_), 2.74 (d, *J* = 10.1, 1H, CHO*H*), 2.09 (t, *J* = 5.3, 1H, CH_2_O*H*), 1.44 (s, 3H, C*H*_3_), 1.39 (s, 3H, C*H*_3_). ^13^C NMR (126 MHz, CDCl_3_): *δ* 144.44 (CH=*C*), 130.25 (*C*H=C), 112.65 (O*C*O), 83.20 (O*C*H–CCH_2_), 77.80 (O*C*H–CHOH), 73.22 (*C*HOH), 59.80 (*C*H_2_OH), 27.54 (*C*H_3_), 26.42 (*C*H_3_). HRMS (ESI+) calcd for C_9_H_14_O_4_Na [M + Na]^+^ 209.0790, found 209.0792. C_9_H_14_O_4_ (186.21): calcd C 58.05, H 7.58; found C 58.21, H 7.46.

#### (−)-(1*R*,4*S*,5*R*)-3-Hydroksymetyl-4,5-*O*-isopropylidenecyclopent-2-en-1-ol ((−)-16)

According to the procedure described above ketoaldehyde (+)-6 (88 mg, 0.48 mmol) was transformed into (−)-16 (83 mg, 92%). [*α*]^20^_D_ = −46.1 (*c* 0.7 in CH_2_Cl_2_). HRMS (ESI+) calcd for C_9_H_14_O_4_Na [M + Na]^+^ 209.0790, found 209.0794. C_9_H_14_O_4_ (186.21): calcd C 58.05, H 7.58; found C 57.98, H 7.69.

#### (+)-(3*S*,4*S*,5*R*)-3-Hydroxy-4,5-*O*-isopropylidenecyclopent-1-ene-1-carbaldehyde ((+)-17)

To a stirred solution of ketoaldehyde (−)-6 (32 mg, 0.175 mmol) and cerium(iii) chloride heptahydrate (92 mg, 0.246 mmol) in methanol (2.5 mL) sodium borohydride (3.3 mg, 0.088 mmol) was added at −90 °C. After 15 min a saturated aqueous solution of NH_4_Cl was added, the cooling batch was removed and the mixture was warmed to room temperature. Methanol was evaporated at the reduced pressure and the residue was extracted with CHCl_3_ (3 × 10 mL). The combined organic extracts were dried over Na_2_SO_4_ and concentrated under vacuum. The crude product was purified by column chromatography (petroleum ether/acetone 4 : 1) to yield hydroxyaldehyde (+)-17 (27 mg, 84%) as a colorless solid. *R*_f_ = 0.36 (petroleum ether/acetone 2 : 1). Mp 101–102 °C. [*α*]^22^_D_ = +101.4 (*c* 0.5 in CH_2_Cl_2_). ^1^H NMR (500 MHz, CDCl_3_): *δ* 9.86 (s, 1H, C*H*O), 6.76 (d, *J* = 1.8, 1H, C*H*=C), 5.26 (d, *J* = 5.6, 1H, OC*H*–CCHO), 4.86 (t, *J* = 5.7, 1H, OC*H*–CHOH), 4.79–4.67 (m, 1H, C*H*OH), 2.94 (d, *J* = 9.6, 1H, O*H*), 1.42 (s, 6H, C*H*_3_). ^13^C NMR (126 MHz, CDCl_3_): *δ* 189.32 (*C*HO), 150.86 (*C*H=C), 144.00 (CH=*C*), 113.18 (O*C*O), 79.95 (O*C*H–C–CHO), 77.17 (O*C*H–CHOH), 73.23 (*C*HOH), 27.20 (*C*H_3_), 25.89 (*C*H_3_). HRMS (ESI+) calcd for C_9_H_12_O_4_Na [M + Na]^+^ 207.0633, found 207.0635. C_9_H_12_O_4_ (184.19): calcd C 58.69, H 6.57; found C 58.80, H 6.60.

#### (−)-Neplanocin A ((−)-NPA)

To a solution of (−)-15 (90 mg, 0.214 mmol) in methanol (3 mL) was added 1 N hydrochloric acid (5 mL) and a mixture was stirred for 3 h at room temperature. The solvents were evaporated under reduced pressure and the crude material was dissolved in methanol and purified by column chromatography using ion exchange resin (Dowex 50 W × 8H^+^) and 0.1 N ammonia solution as eluent to yield (−)-NPA (36 mg, 94%) as a colorless crystal solid. Mp 214–217 °C. [*α*]^25^_D_ = −154.0 (*c* 0.4 in H_2_O). ^1^H NMR (600 MHz, DMSO-*d*_6_): *δ* 8.13 (s, 1H, C*H*=N (adenine)), 8.07 (s, 1H, C*H*=N (adenine)), 7.21 (s, 2H, N*H*_2_), 5.70 (s, 1H, C*H*=C), 5.35 (s, 1H, C*H*N), 5.16 (d, *J* = 6.8, 1H, CHO*H*), 4.97 (d, *J* = 6.0, 1H, CHO*H*), 4.92 (t, *J* = 5.4, 1H, CH_2_O*H*), 4.43 (d, *J* = 5.5, 1H, OC*H*–C–N), 4.31 (t, *J* = 5.5, 1H, OC*H*–C–CH_2_), 4.17–4.07 (m, 2H, C*H*_2_OH). ^13^C NMR (151 MHz, DMSO-*d*_6_): *δ* 155.90 (*C*H=N (adenine)), 152.33 (*C*=C (adenine)), 149.98 (C=*C* (adenine)), 149.66 (C=*C*–CH_2_), 139.54 (*C*H=N (adenine)), 123.48 (*C*HN), 119.13 (*C*–NH_2_ (adenine)), 76.45 (O*C*H–CCH_2_), 72.12 (O*C*H–CCH_2_), 64.19 (*C*=CCH_2_), 58.45 (*C*H_2_). C_11_H_13_N_5_O_3_ (263.25): calcd C 50.19, H 4.98; found C 50.24, H 4.80.

#### (+)-Neplanocin A ((+)-NPA)

By analogy to the above procedure (+)-15 (97 mg, 0.232 mmol) was transformed into (+)-NPA in 92% yield. Mp 212–215 °C. [*α*]^25^_D_ = +152.5 (*c* 0.5 in H_2_O). C_11_H_13_N_5_O_3_ (263.25): calcd C 50.19, H 4.98; found C 50.30, H 4.84.

### Cell cultures

The following cell lines were used in the research: MOLT-4 (T-cell acute lymphoblastic leukemia), U87-MG (human glioblastoma), MDA-MB-231 (human breast adenocarcinoma), A431 (epidermoid carcinoma), MCF-7 (breast cancer) and normal human fibroblasts. The MOLT-4 and U87-MG cells were purchased from the European Collection of Authenticated Cell Cultures (ECACC, Salisbury, UK), the MDA-MB-231 cells were purchased from Cell Biolabs (San Diego, California, USA), the A431 and MCF-7 cell lines were purchased from American Type Culture Collection (ATCC, Manassas, Virginia, USA), the human fibroblasts were purchased from Celther Polska (Lodz, Poland). All the cell lines were maintained at 37 °C in an atmosphere of 5% CO_2_ in the medium appropriate for the cell type. The MCF-7 and A431 cells were cultured in Dulbecco's modified Eagle's medium (Sigma-Aldrich, St. Louis, MO) supplemented with 10% fetal bovine serum (FBS) (Sigma-Aldrich, St. Louis, MO), MOLT-4 in RPMI 1640 medium (Sigma-Aldrich, St. Louis, MO) with 10% FBS, U87-MG in MEM medium containing 10% FBS, MDA-MB-231 cells in DMEM medium with 10% FBS supplemented with l-glutamine and non-essential amino acids. All media were enriched in antibiotics – 100 U per L penicillin G and 100 U per L streptomycin.

### Cell viability test

The cells in the exponential growth phase were seeded in a complete medium at a concentration of 7000 cells per well on 96-well plates and were allowed to adhere for 24 h. The number of cells was evaluated using Scepter™ 2.0 Cell Counter (Merck, Darmstadt, Germany). The tested compounds were added in the concentration range from 1 to 1000 μM. The final concentration of DMSO in the cell culture medium was 1%. After 48 hours of incubation at 37 °C in an atmosphere of 5% CO_2_, the number of living cells in the culture was evaluated. The cytotoxicity of all compounds was determined by the MTT [3-(4,5-dimethylthiazol-2-yl)-2,5-diphenyltetrazolium bromide; Sigma, St. Louis, MO] assay as described previously.^61^ Each viability point represents the mean ± SE from at least three independent experiments performed thrice. The IC_50_ values were calculated from the dose–response curves.

### DAPI staining

The A431 cells were seeded into a 48-well plate at a concentration of 500 000 cells per well and cultured at 37 °C for 24 hours. After that, the culture medium from each well was changed to a new one containing tested compounds at a concentration of 100 μM. After 18 hours incubation, cell were washed thrice with PBS buffer and fixed using 3.8% paraformaldehyde. After repeated washing, cells were stained for 10 min with DAPI (Sigma-Aldrich, St. Louis, MO) at a final concentration of 5 μg mL^−1^ and then washed three times with PBS. Microscopic observation was performed using Nikon Eclipse microscope with appropriate optical filters. The normal, apoptotic and dividing nuclei from at least 6 fields per sample were counted and the percentage of each type of cells were calculated. Visualization of the cells were performed using NisElement (Nikon). The images were analyzed with ImageJ software.

### Caspase-3/7 assay

The level of caspases activity was measured using the Apo-ONE homogeneous caspase-3/7 assay (Promega, USA) according to the manufacturer's instructions. The A431 cells were seeded into 96-well black plates in an amount of 20 000 cells per well and allowed to adhere for the next 24 hours. Then, grow medium was changed for the new one containing tested compounds at the concentration of 100 μM. The cells treated only with DMSO instead of the tested compounds were used as a control. Staurosporin (5 μM) – known inducer of apoptosis, was used as a positive control. After 18 hours incubation in 5% CO_2_ at 37 °C, microscopic observation of cell's morphology was performed. Then, the profluorescent caspase substrate in optimized bifunctional cell lysis/activity buffer was added to the wells. Incubation was carried out for the next 6 hours in the dark. Afterwards, the caspase-3/7 enzymatic activity was assessed based on the level of fluorescent signals. Fluorescence measurement was performed using a Synergy HT plate reader (BIO-TEK). The caspase activity was calculated based on the relative fluorescent units (RFU) according to the formula: [(RFU of the sample − RFU of blank wells)/RFU of the control] × 100%.

## Conflicts of interest

There are no conflicts of interest to declare.

## Supplementary Material

RA-010-D0RA06394K-s001
