# Supplementary material for: A novel route to a chiral building block for the preparation of cyclopentenyl carbocyclic nucleosides. Synthesis and anticancer activity of enantiomeric neplanocins A
Source: RSC Adv. 2020 Aug 27;10(53):31838–47. doi: 10.1039/d0ra06394k (PMC9056547; doi:10.1039/d0ra06394k)
Supplement: RA-010-D0RA06394K-s001 [file RA-010-D0RA06394K-s001.pdf]

Electronic Supplementary Material (ESI) for RSC Advances.
This journal is © The Royal Society of Chemistry 2020

SUPPORTING INFORMATION

A novel route to the chiral building block for the preparation of cyclopentenyl carbocyclic nucleosides. Synthesis and anticancer activity of enantiomeric neplanocins A

Beata Łukasik,[a] Maciej Mikina,[a] Marian Mikołajczyk,[a] Róża Pawłowska,*[b] and Remigiusz Żurawiński*[a]

[a] *Division of Organic Chemistry, Centre of Molecular and Macromolecular Studies, Polish Academy of Sciences, Sienkiewicza 112, 90-363 Łódź, Poland, Fax: (+48)-426803260, E-mail: remzur@cbmm.lodz.pl*
[b] *Division of Bioorganic Chemistry, Centre of Molecular and Macromolecular Studies, Polish Academy of Sciences, Sienkiewicza 112, 90-363 Łódź, Poland*

Table of Contents

^{1}H and ^{13}C spectra of compounds **3c**, **13-17** and neplanocin A **(NPA).**

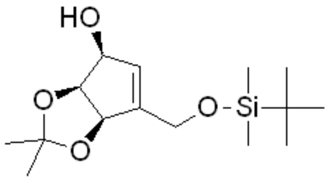
HO
O
O
O-Si

3c

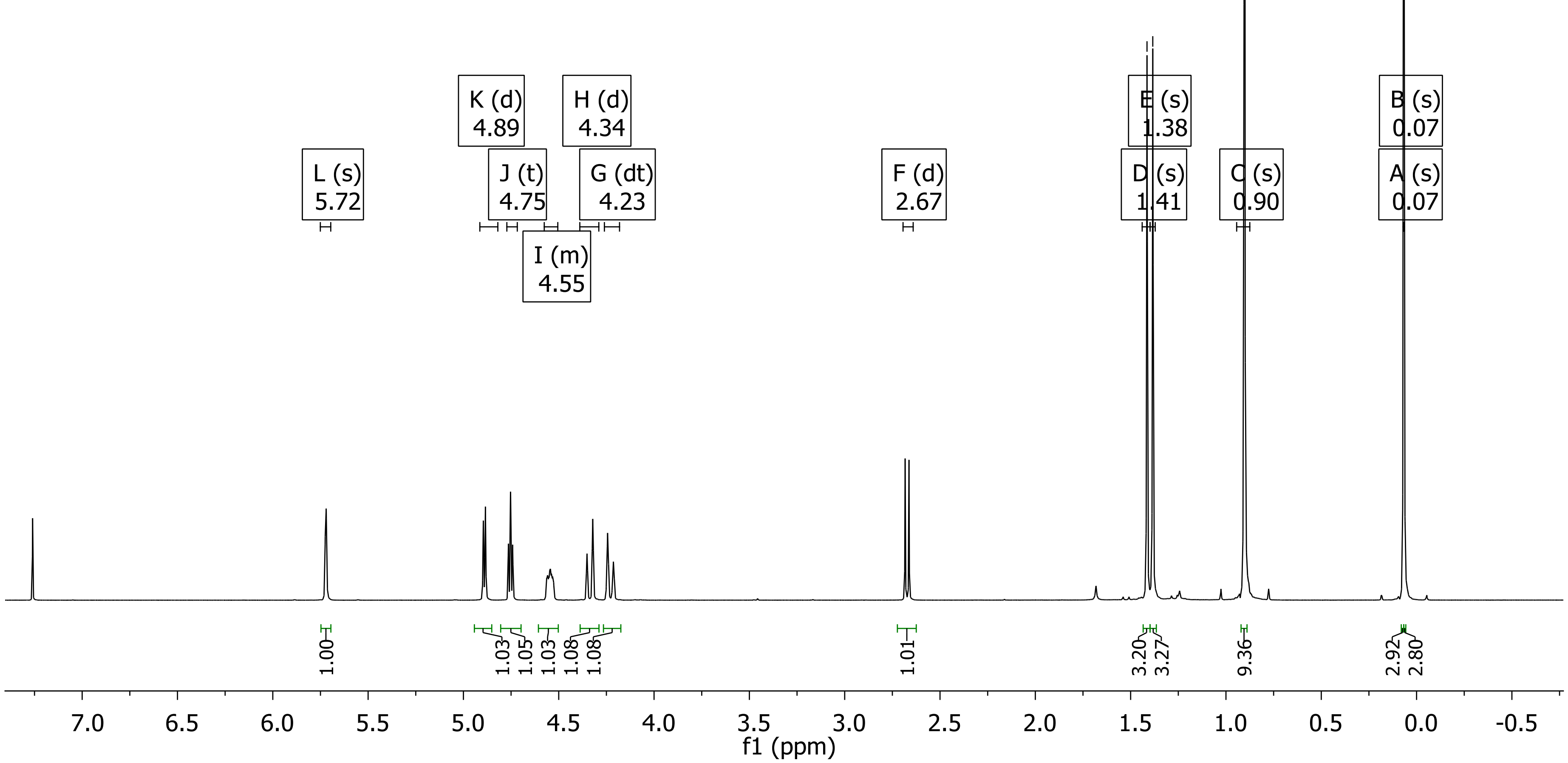
1.41
1.38
0.90
0.07
0.07
K (d) 4.89
H (d) 4.34
E (s) 1.38
B (s) 0.07
L (s) 5.72
J (t) 4.75
G (dt) 4.23
F (d) 2.67
D (s) 1.41
C (s) 0.90
A (s) 0.07
I (m) 4.55
1.00
1.03
1.05
1.03
1.08
1.08
1.01
3.20
3.27
9.36
2.92
2.80
7.0 6.5 6.0 5.5 5.0 4.5 4.0 3.5 3.0 2.5 2.0 1.5 1.0 0.5 0.0 -0.5
f1 (ppm)

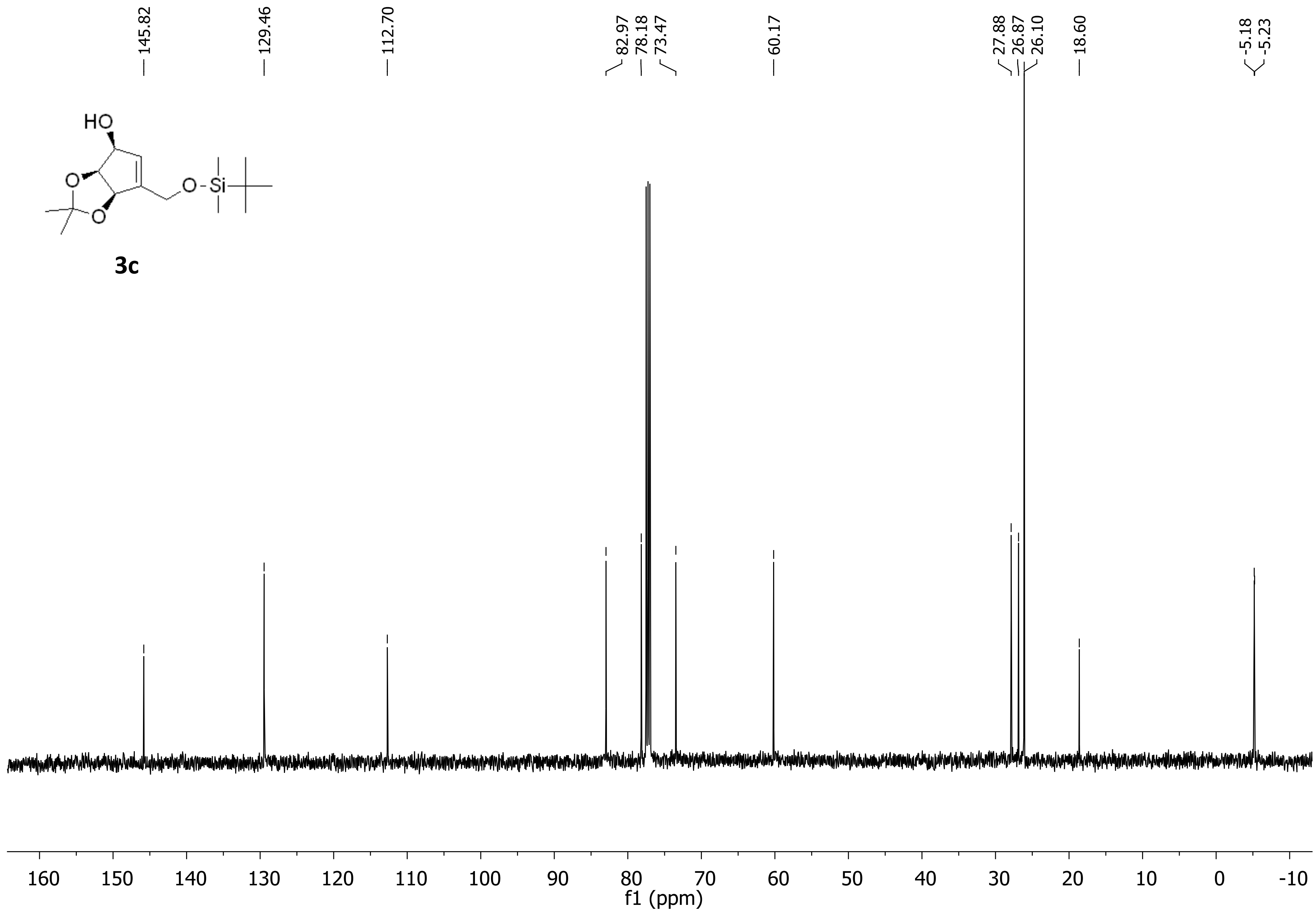
145.82
129.46
112.70
82.97
78.18
73.47
60.17
27.88
26.87
26.10
18.60
-5.18
-5.23
HO
O
O
O
Si
3c
160 150 140 130 120 110 100 90 80 70 60 50 40 30 20 10 0 -10
f1 (ppm)

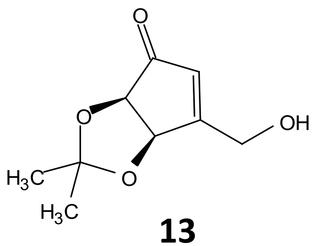
O
OH
O
O
H3C
H3C

13

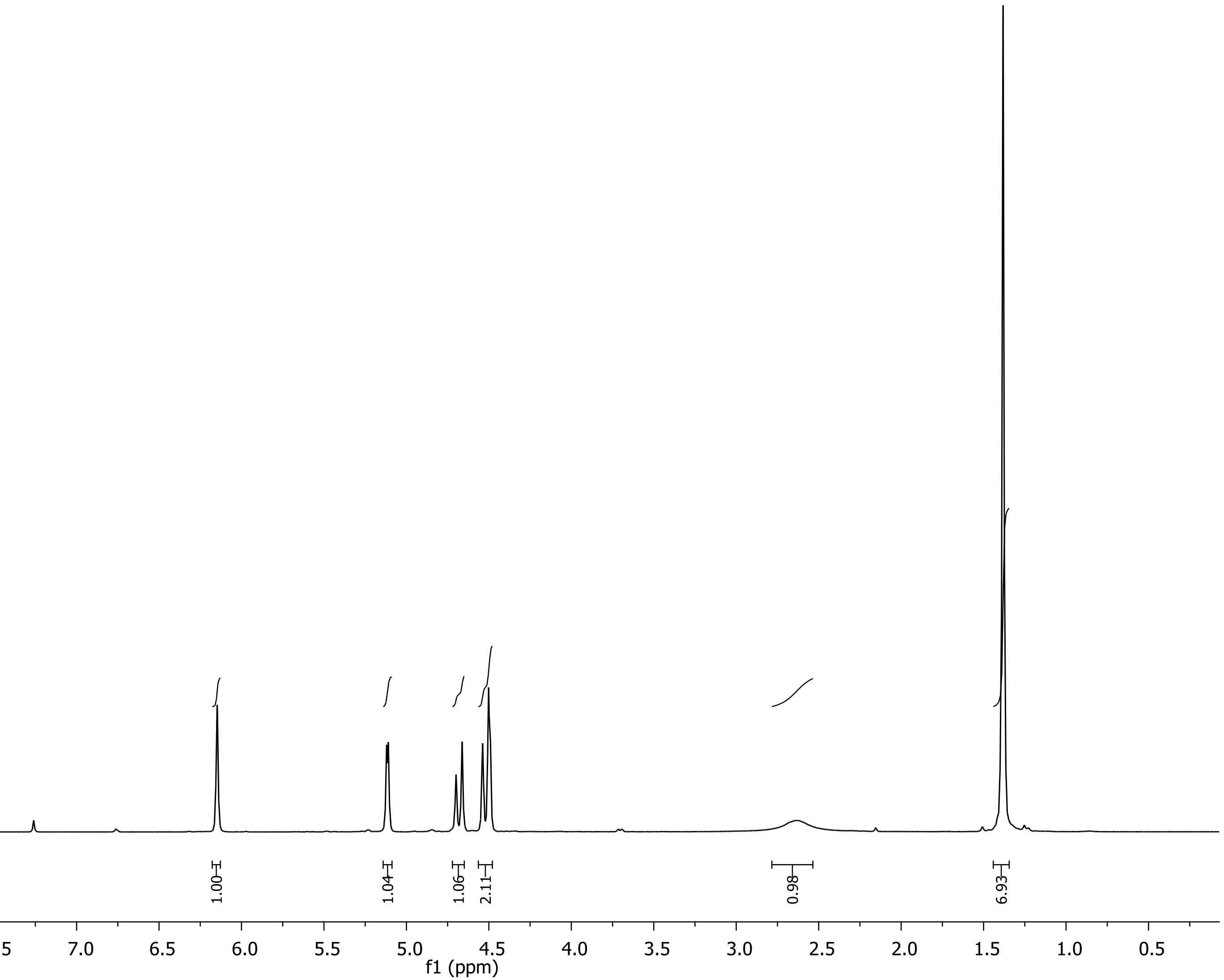
1.00
1.04
1.06
2.11
0.98
6.93
9.0
8.5
8.0
7.5
7.0
6.5
6.0
5.5
5.0
4.5
4.0
3.5
3.0
2.5
2.0
1.5
1.0
0.5
f1 (ppm)

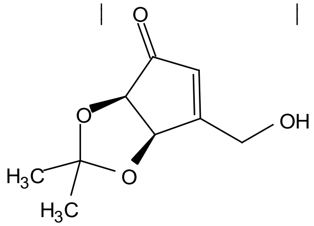
O
O
O
OH
H3C
H3C

13

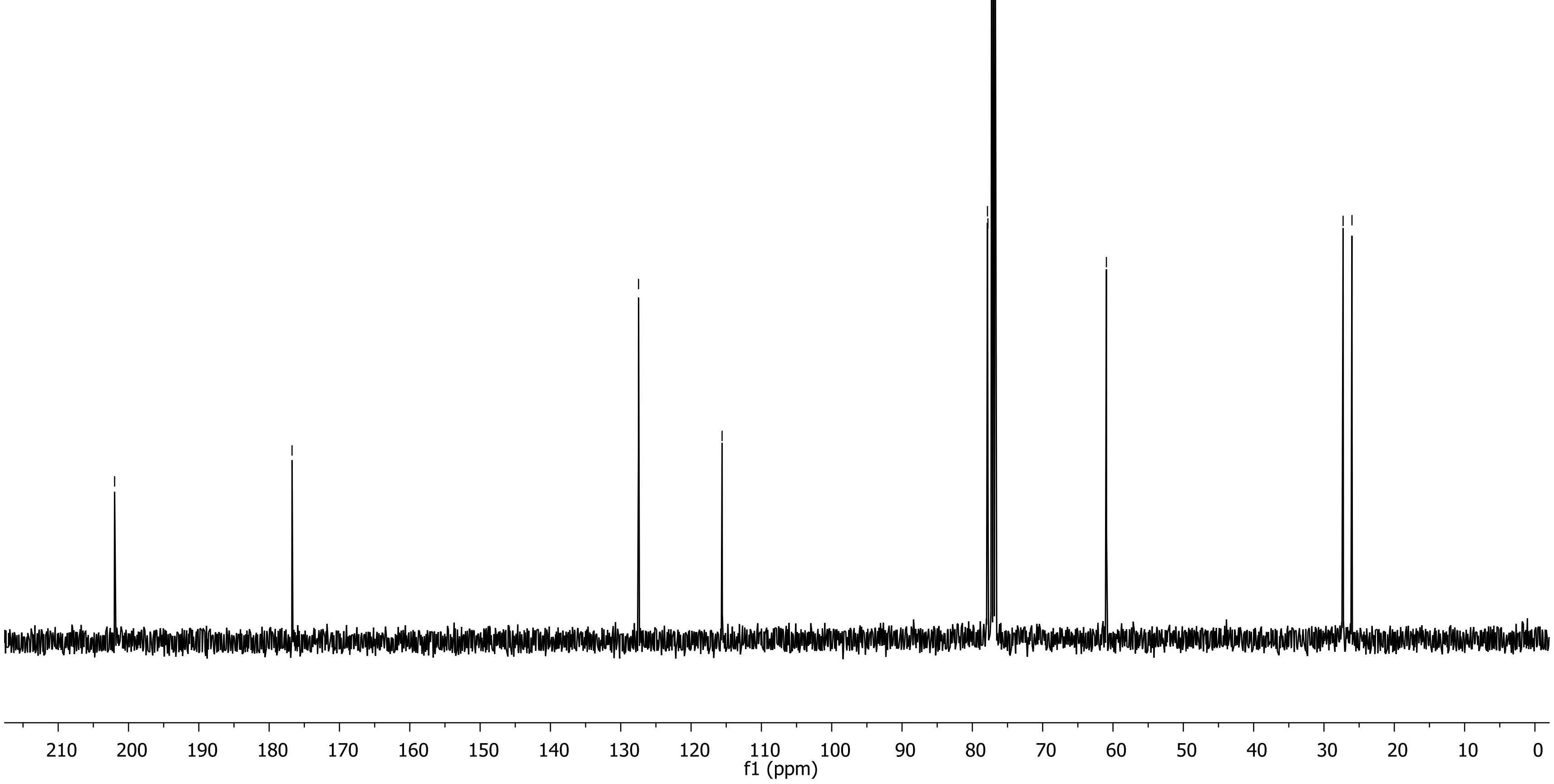
201.97
176.74
127.47
115.59
77.85
77.79
60.94
27.28
26.02
210
200
190
180
170
160
150
140
130
120
110
100
90
80
70
60
50
40
30
20
10
0
f1 (ppm)

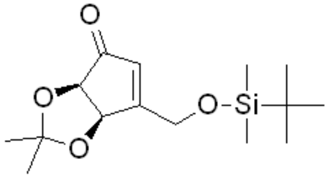

14

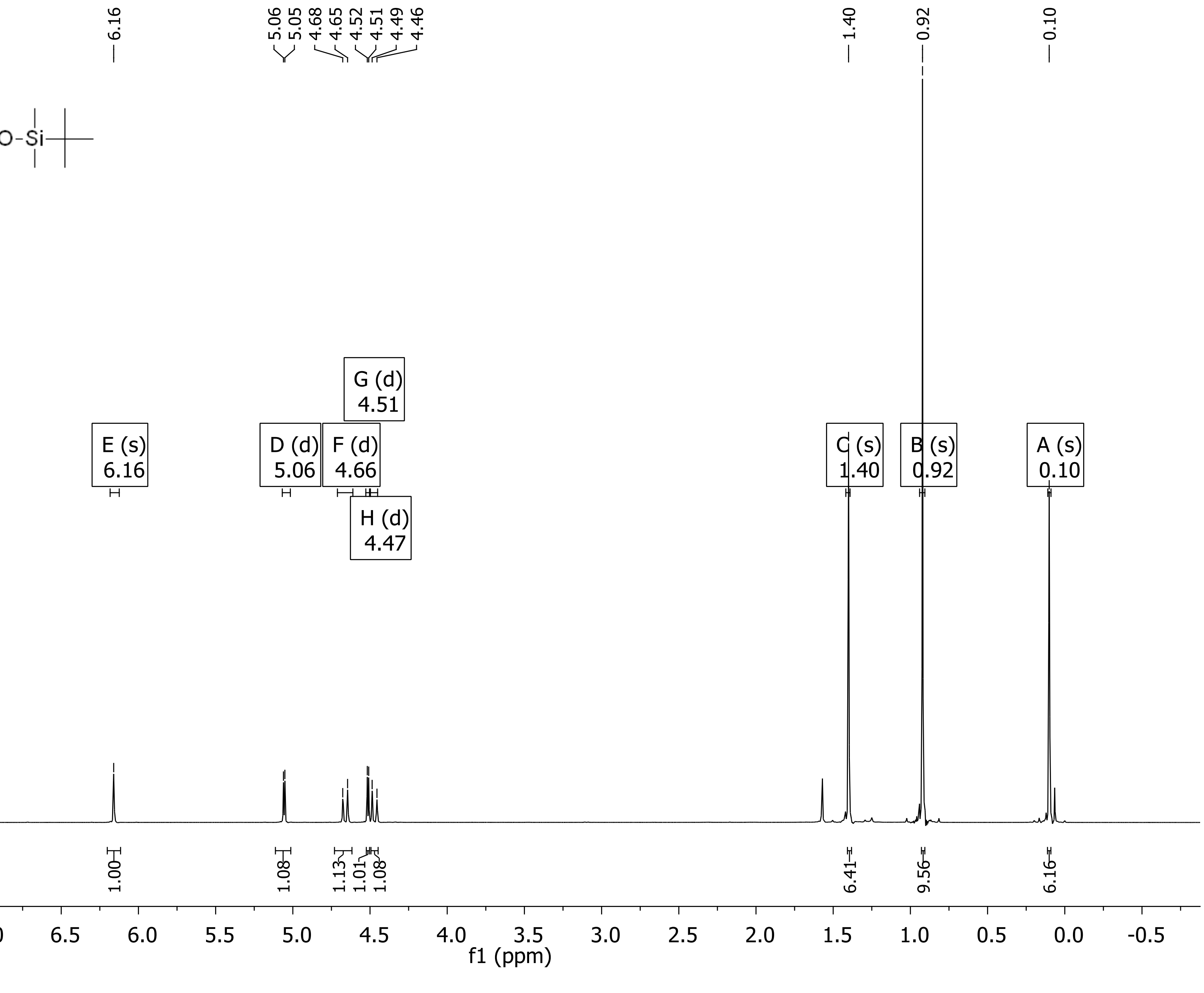

6.16
5.06
5.05
4.68
4.65
4.52
4.51
4.49
4.46
1.40
0.92
0.10
G (d) 4.51
E (s) 6.16
D (d) 5.06
F (d) 4.66
H (d) 4.47
C (s) 1.40
B (s) 0.92
A (s) 0.10
1.00
1.08
1.13
1.01
1.08
6.41
9.56
6.16
8.0 7.5 7.0 6.5 6.0 5.5 5.0 4.5 4.0 3.5 3.0 2.5 2.0 1.5 1.0 0.5 0.0 -0.5
f1 (ppm)

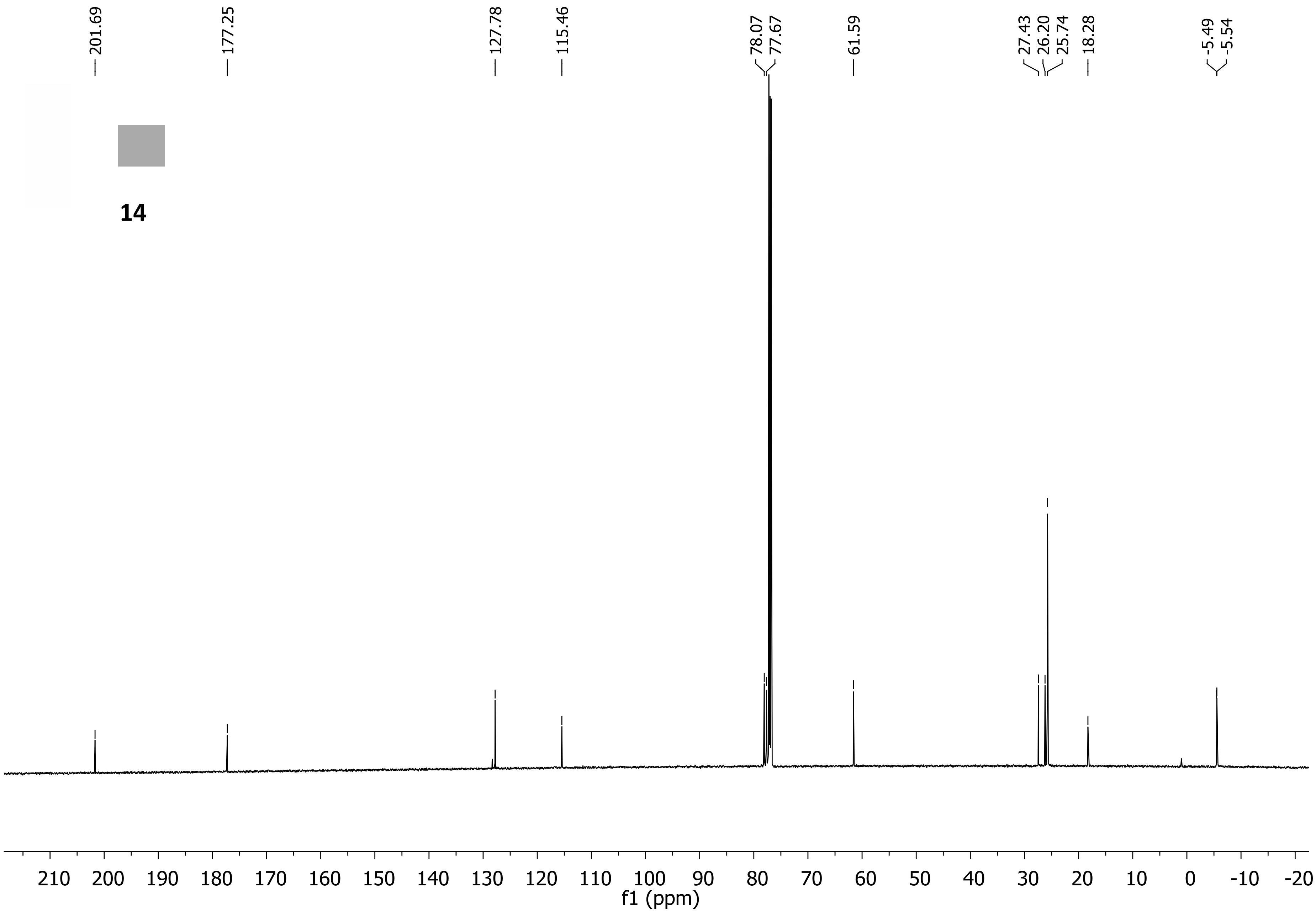
201.69
177.25
127.78
115.46
78.07
77.67
61.59
27.43
26.20
25.74
18.28
-5.49
-5.54
210 200 190 180 170 160 150 140 130 120 110 100 90 80 70 60 50 40 30 20 10 0 -10 -20
f1 (ppm)

14

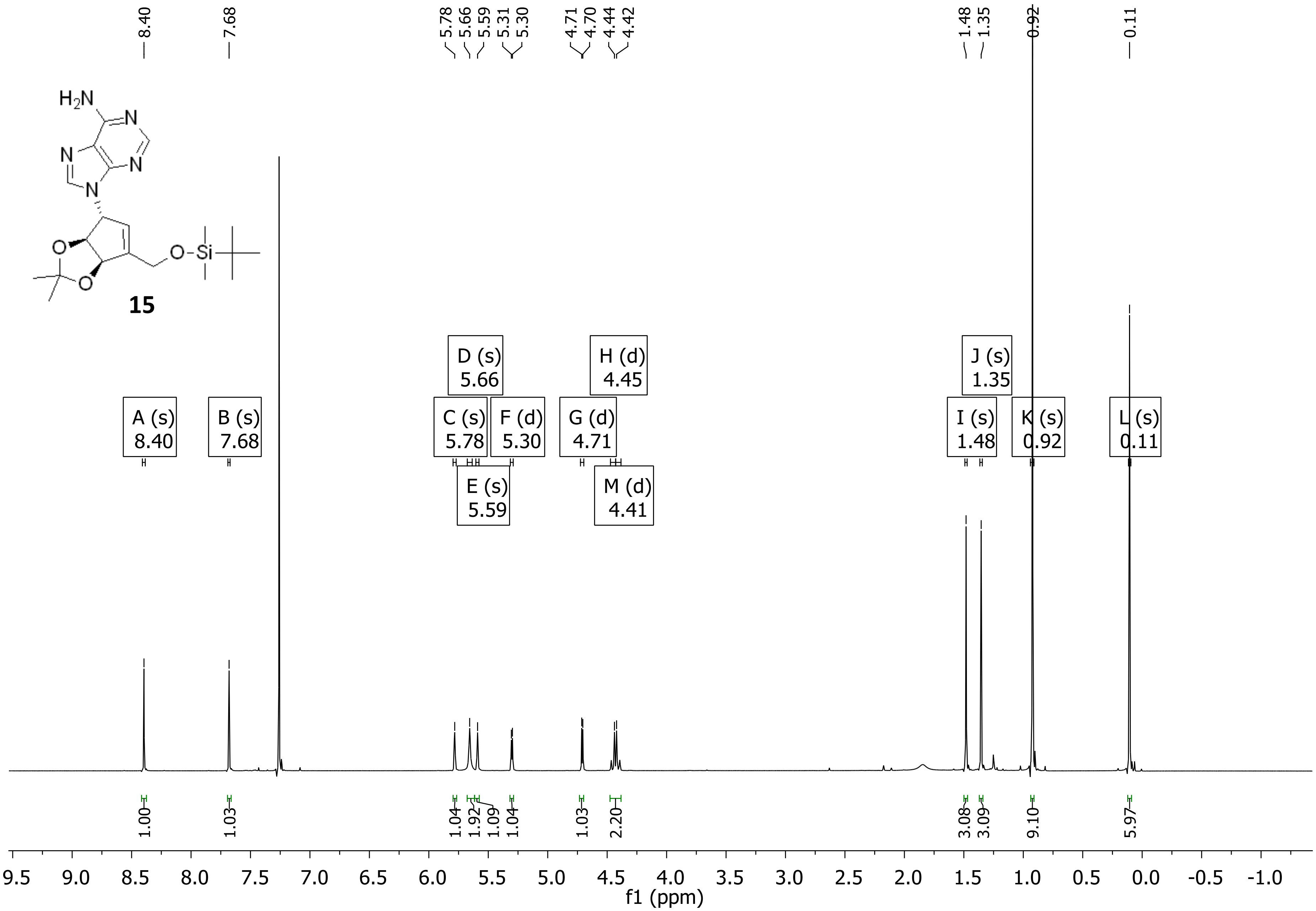

8.40
7.68
5.78
5.66
5.59
5.31
5.30
4.71
4.70
4.44
4.42
1.48
1.35
0.92
0.11
H_2N
N
N
N
N
O
O
O
Si
15
D (s) 5.66
H (d) 4.45
J (s) 1.35
A (s) 8.40
B (s) 7.68
C (s) 5.78
F (d) 5.30
G (d) 4.71
I (s) 1.48
K (s) 0.92
L (s) 0.11
E (s) 5.59
M (d) 4.41
1.00
1.03
1.04
1.92
1.09
1.04
1.03
2.20
3.08
3.09
9.10
5.97
9.5 9.0 8.5 8.0 7.5 7.0 6.5 6.0 5.5 5.0 4.5 4.0 3.5 3.0 2.5 2.0 1.5 1.0 0.5 0.0 -0.5 -1.0
f1 (ppm)

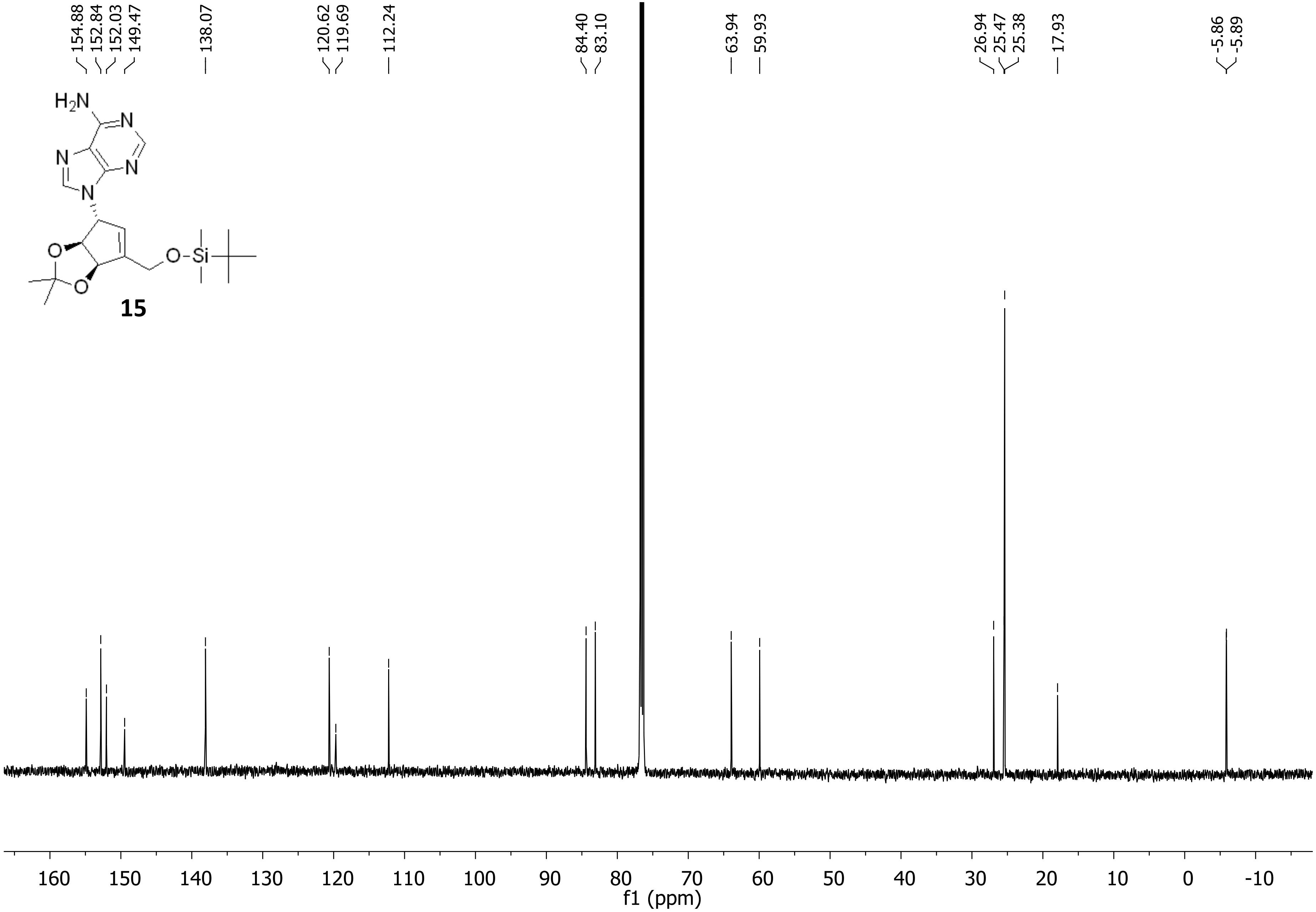
154.88
152.84
152.03
149.47
138.07
120.62
119.69
112.24
84.40
83.10
63.94
59.93
26.94
25.47
25.38
17.93
-5.86
-5.89
H_2N
N
N
N
N
O
O
O
Si
15
160 150 140 130 120 110 100 90 80 70 60 50 40 30 20 10 0 -10
f1 (ppm)

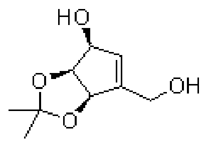
HO
O
O
OH

16

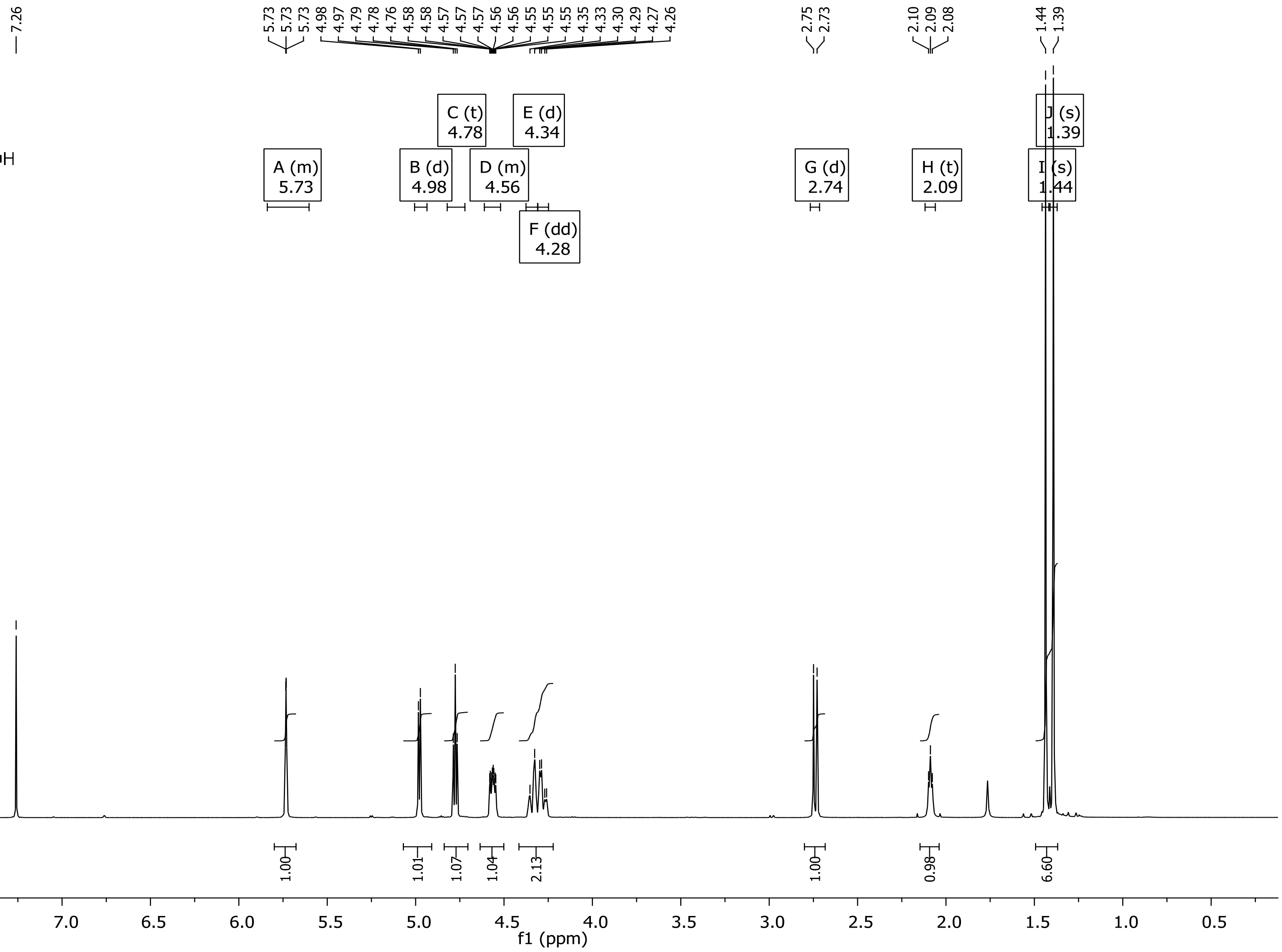
7.26
5.73 5.73 5.73 4.98 4.97 4.79 4.78 4.76 4.58 4.58 4.57 4.57 4.56 4.56 4.55 4.55 4.55 4.35 4.33 4.30 4.29 4.27 4.26
2.75 2.73
2.10 2.09 2.08
1.44 1.39
A (m) 5.73
B (d) 4.98
C (t) 4.78
D (m) 4.56
E (d) 4.34
F (dd) 4.28
G (d) 2.74
H (t) 2.09
I (s) 1.44
J (s) 1.39
1.00
1.01
1.07
1.04
2.13
1.00
0.98
6.60
8.0 7.5 7.0 6.5 6.0 5.5 5.0 4.5 4.0 3.5 3.0 2.5 2.0 1.5 1.0 0.5
f1 (ppm)

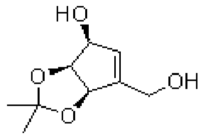
HO
O
O
OH

16

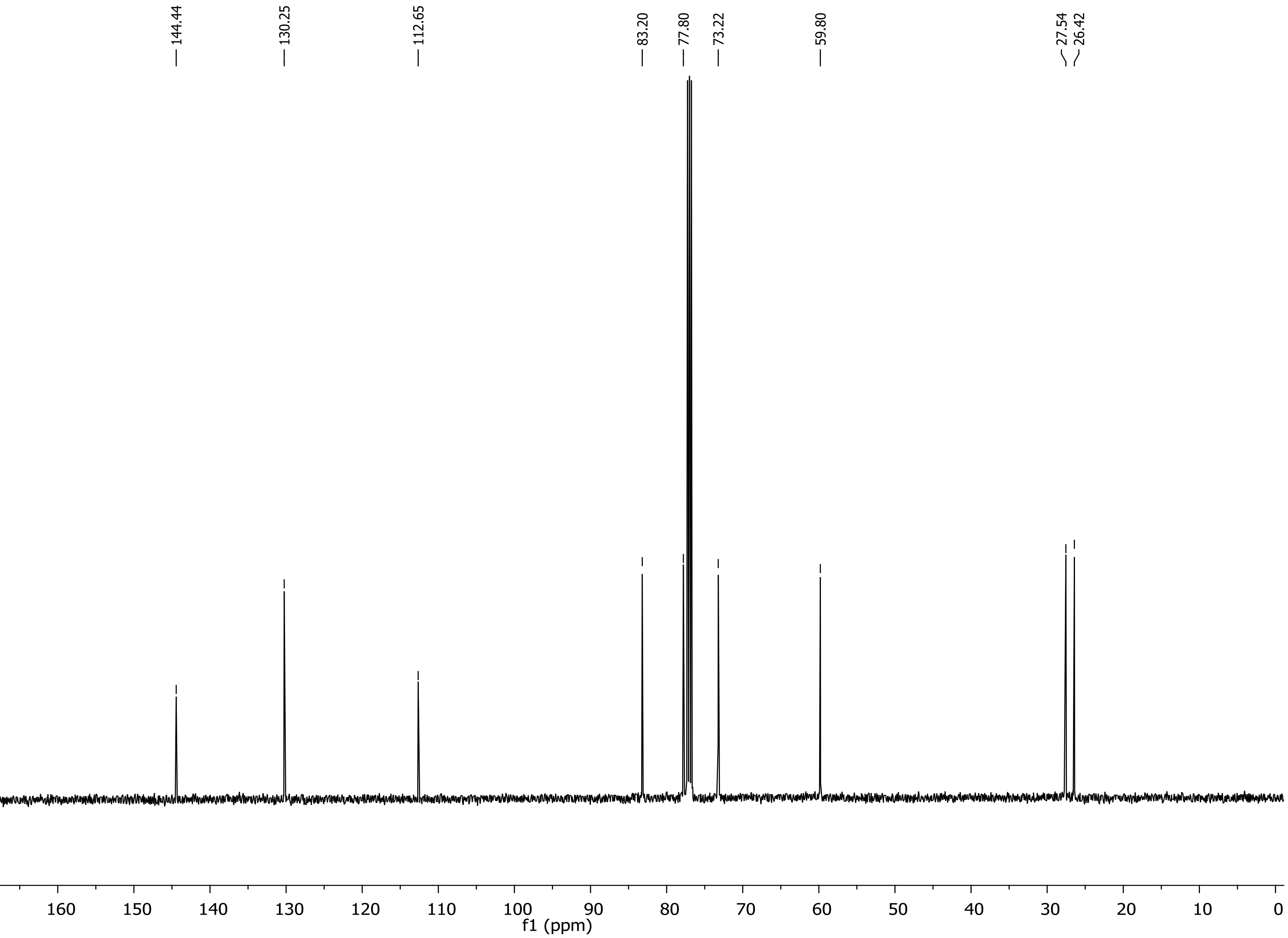
144.44
130.25
112.65
83.20
77.80
73.22
59.80
27.54
26.42
190
180
170
160
150
140
130
120
110
100
90
80
70
60
50
40
30
20
10
0
f1 (ppm)

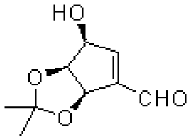

17

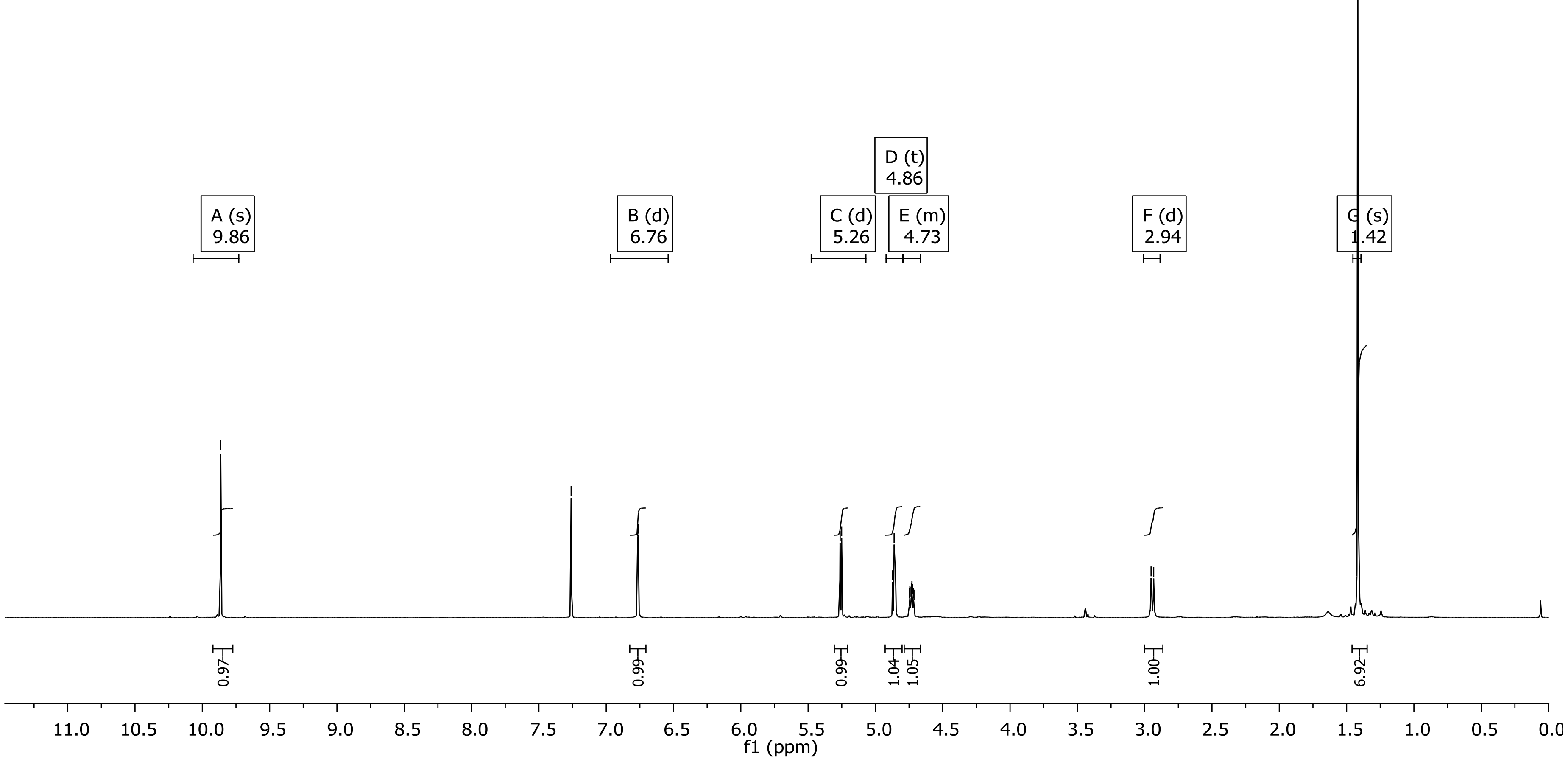

9.86
7.26
6.77
6.76
5.26
5.25
4.87
4.86
4.85
4.75
4.74
4.74
4.73
4.73
4.73
4.72
4.71
2.95
2.93
1.42
A (s) 9.86
B (d) 6.76
C (d) 5.26
D (t) 4.86
E (m) 4.73
F (d) 2.94
G (s) 1.42
0.97
0.99
0.99
1.04
1.05
1.00
6.92
11.0
10.5
10.0
9.5
9.0
8.5
8.0
7.5
7.0
6.5
6.0
5.5
5.0
4.5
4.0
3.5
3.0
2.5
2.0
1.5
1.0
0.5
0.0
f1 (ppm)

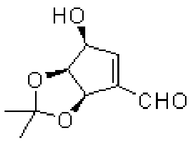
HO
O
O
CHO

17

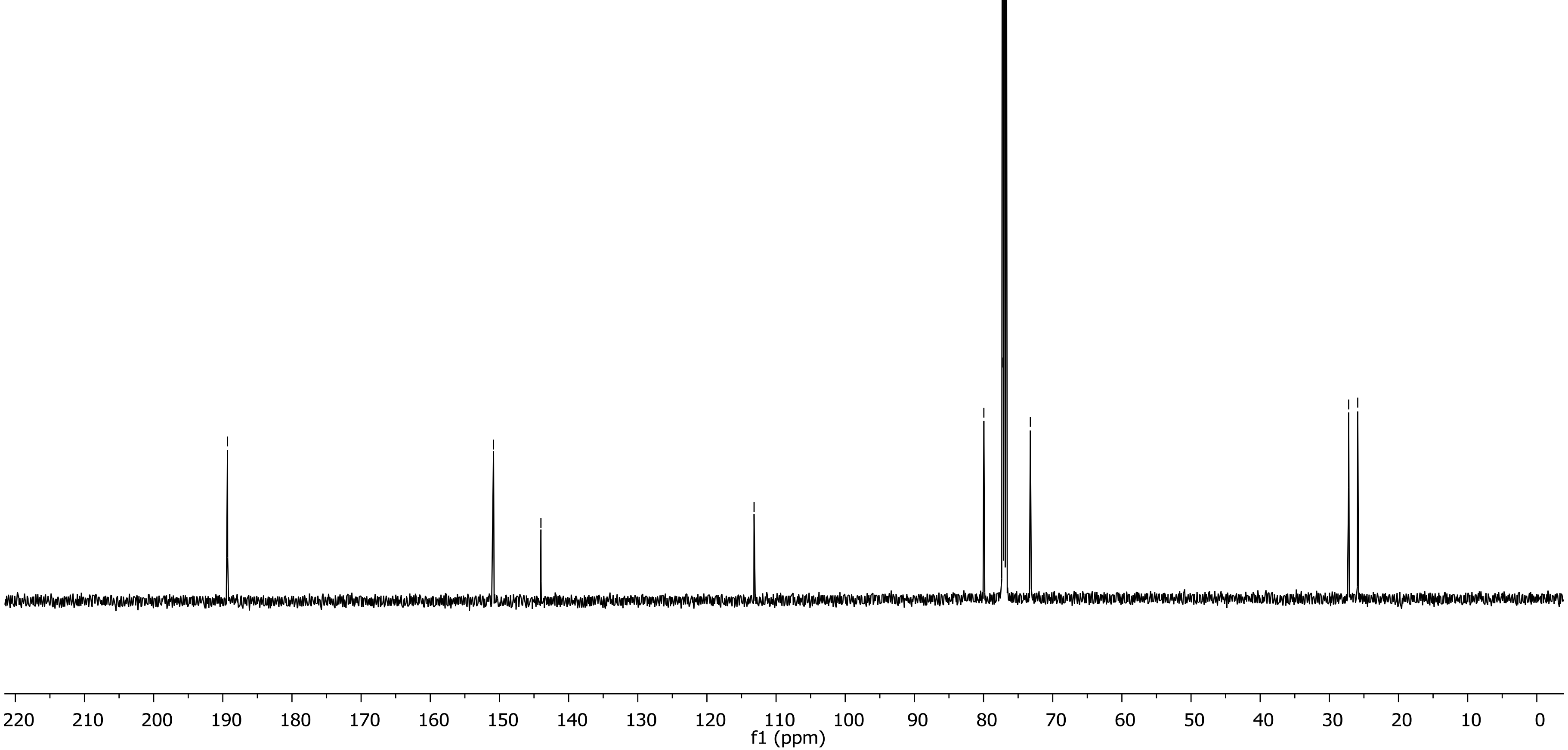
189.32
150.86
144.00
113.18
79.95
77.17
77.00
73.23
27.20
25.89
220 210 200 190 180 170 160 150 140 130 120 110 100 90 80 70 60 50 40 30 20 10 0
f1 (ppm)

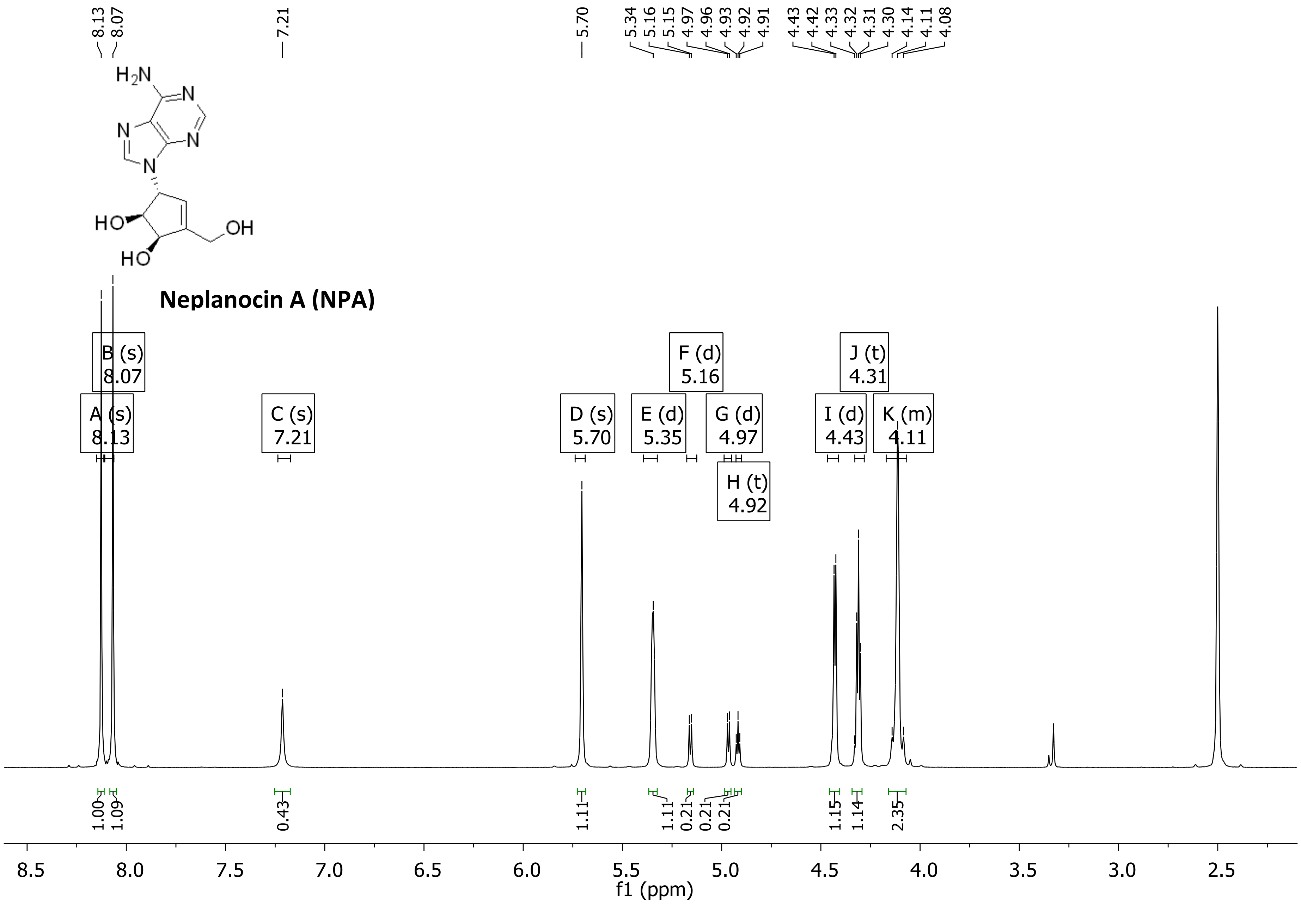
8.13
8.07
7.21
5.70
5.34
5.16
5.15
4.97
4.96
4.93
4.92
4.91
4.43
4.42
4.33
4.32
4.31
4.30
4.14
4.11
4.08
H2N
N
N
N
N
HO
HO
OH
Neplanocin A (NPA)
B (s) 8.07
A (s) 8.13
C (s) 7.21
D (s) 5.70
E (d) 5.35
F (d) 5.16
G (d) 4.97
H (t) 4.92
I (d) 4.43
J (t) 4.31
K (m) 4.11
1.00
1.09
0.43
1.11
1.11
0.21
0.21
0.21
1.15
1.14
2.35
8.5
8.0
7.5
7.0
6.5
6.0
5.5
5.0
4.5
4.0
3.5
3.0
2.5
f1 (ppm)

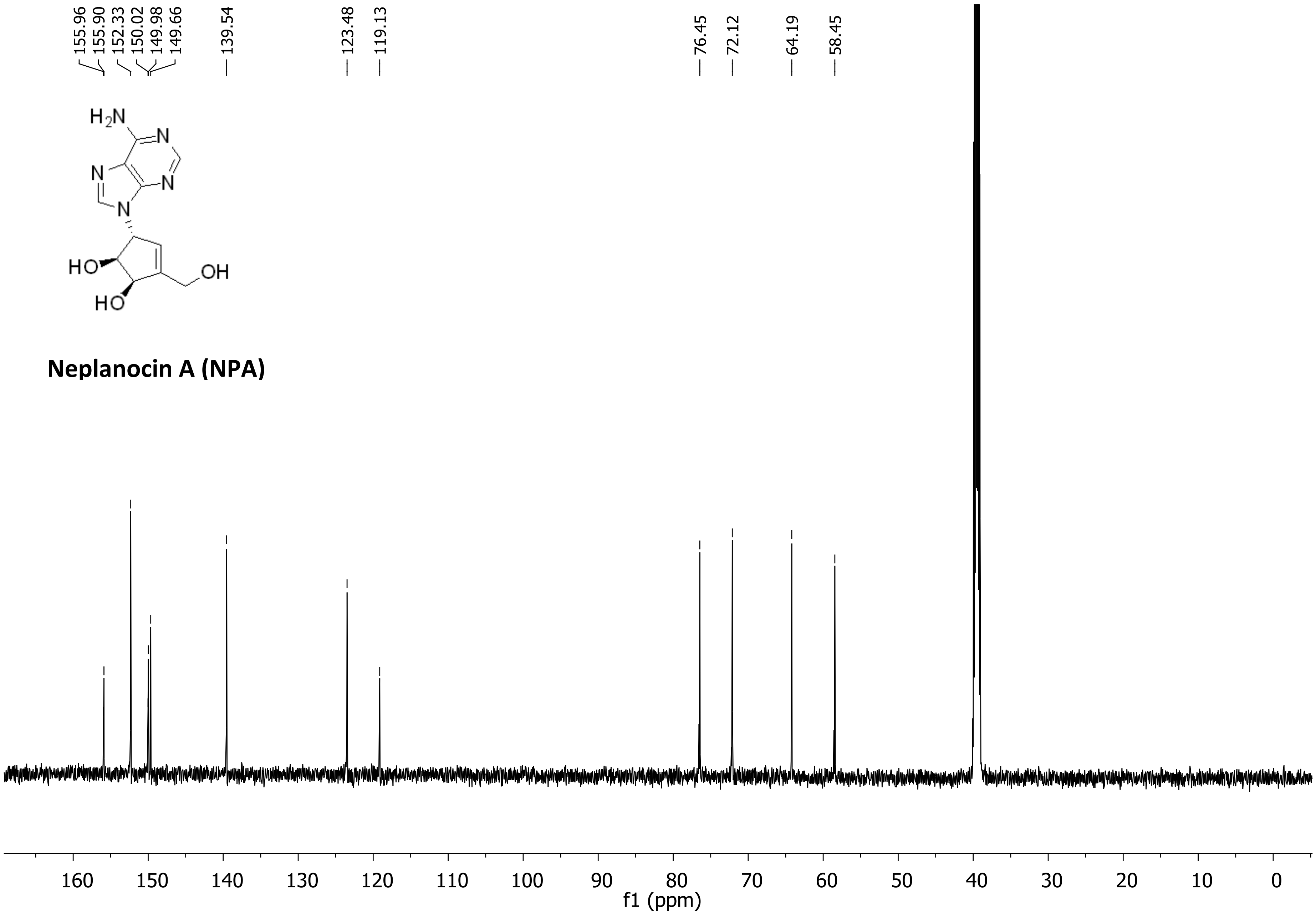

Neplanocin A (NPA)
